# Simultaneous manifestation of metallic conductivity and single-molecule magnetism in a layered molecule-based compound†

**DOI:** 10.1039/d0sc04040a

**Published:** 2020-09-01

**Authors:** Yongbing Shen, Hiroshi Ito, Haitao Zhang, Hideki Yamochi, Seiu Katagiri, Shinji K. Yoshina, Akihiro Otsuka, Manabu Ishikawa, Goulven Cosquer, Kaiji Uchida, Carmen Herrmann, Takefumi Yoshida, Brian K. Breedlove, Masahiro Yamashita

**Affiliations:** Department of Chemistry, Graduate School of Science, Tohoku University Sendai Japan shenyongbing17@gmail.com; Department of Applied Physics, Nagoya University Chikusa-ku Nagoya 464-603 Japan; Institute of Inorganic and Applied Chemistry, University of Hamburg Martin-Luther-King-Platz 6 20146 Hamburg Germany; Division of Chemistry, Graduate School of Science, Kyoto University Sakyo-ku Kyoto 606-8502 Japan; Research Center for Low Temperature and Materials Sciences, Kyoto University Sakyo-ku Kyoto 606-8501 Japan; Research Group of Solid Material Chemistry, Graduate School of Science, Hiroshima University 1-3-1 Kagamiyama, Higashihiroshima Hiroshima 739-8526 Japan; School of Materials Science and Engineering, Nankai University Tianjin 300350 China

## Abstract

Single-molecule magnets (SMMs) show superparamagnetic behaviour below blocking temperature at the molecular scale, so they exhibit large magnetic density compared to the conventional magnets. Combining SMMs and molecular conductors in one compound will bring about new physical phenomena, however, the synergetic effects between them still remain unexplored. Here we present a layered molecule-based compound, β′′-(BEDO-TTF)_4_ [Co(pdms)_2_]·3H_2_O (**BO4**), (BEDO-TTF (BO) and H_2_pdms are bis(ethylenedioxy)tetrathiafulvalene and 1,2-bis(methanesulfonamido)benzene, respectively), which was synthesized by using an electrochemical approach and studied by using crystal X-ray diffraction. This compound simultaneously exhibited metallic conductivity and SMM behaviour up to 11 K for the first time. The highest electrical conductivity was 400–650 S cm^−1^ at 6.5 K, which is the highest among those reported so far for conducting SMM materials. Furthermore, antiferromagnetic ordering occurred below 6.5 K, along with a decrease in conductivity, and the angle-independent negative magnetoresistance suggested an effective electron correlation between the conducting BO and Co(pdms)_2_ SMM layers (d–π interactions). The strong magnetic anisotropy and two-dimensional conducting plane play key roles in the low-temperature antiferromagnetic semiconducting state. **BO4** is the first compound exhibiting antiferromagnetic ordering among SMMs mediated by π-electrons, demonstrating the synergetic effects between SMMs and molecular conductors.

## Abstract 2

A metallic single-molecule magnet was synthesised demonstrating simultaneous metallic conduction and excellent SMM properties at the same temperature range for the first time, with potential applications in molecule-based quantum spintronics.

## Graphical abstract

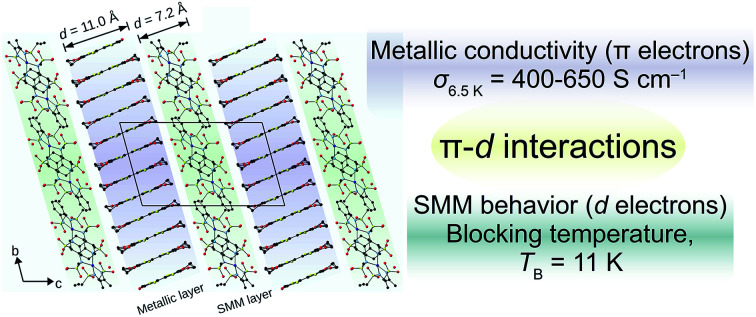

## Introduction

Exploitation of multifunctionalities on the nanoscale is a critical challenge in modern chemistry to satisfy the rapidly growing demands in electronics.^1^ Combining both electrical conductivity (*σ*) and magnetism in one compound to afford bifunctional materials has been intensively studied by P. Day and H. Kobayashi *et al.*^2^ The discovery of the giant magnetoresistance (GMR) effect by A. Fert *et al.*^3^ and P. Grünberg *et al.*^4^ in 1988 established the area of spintronics and significantly improved the size and storage capacity of magnetic digital data drives in the 21st century. The research in this field has concentrated on combining molecular conductors, such as bis(ethylenedithio)tetrathiafulvalene (BEDT-TTF),^5^ 7,7,8,8-tetracyanoquinodimethane (TCNQ)^6^ and various single-molecule magnets (SMMs), such as [Mn_2_^III^(5-MeOsaltmen)_2_]^−^ and [Dy(CF_3_COO)_4_]^−^,^5,7^ where 5-MeOsaltmen is the *N*,*N*′-(1,1,2,2-tetramethylethylene)-bis(5-methoxysalicylideneiminato) dianion. SMMs can magnetically store information at a molecular level.^8,9^ Thus, they are good candidates for use in molecular spintronic devices, like spin-valves, and due to their long relaxation times, they are applied in quantum computers.^10^ Moreover, the combination of SMMs and electrical conductors may allow for nanoscale functionality beyond Moore's limitation.^11^

The construction of conducting SMMs has been examined;^12^ however, so far obtained materials exhibited semiconductor behaviour and SMM behaviour in different temperature ranges. They conduct above liquid nitrogen temperature and behave as SMMs at liquid helium temperature, and no synergism has been observed. Therefore, the search for suitable molecular conductors and SMMs has been intensively pursued. Recently, our group has reported (TTF)_2_[Co(pdms)_2_], which behaves as a semiconductor and an SMM in the same temperature region.^13^ Due to its unusual mixed stacking structure, strong charge transfer between TTF and [Co(pdms)_2_] affords electrical conduction down to 2 K with SMM properties up to 5 K, however, it is not metallic.

**Fig. 1:**
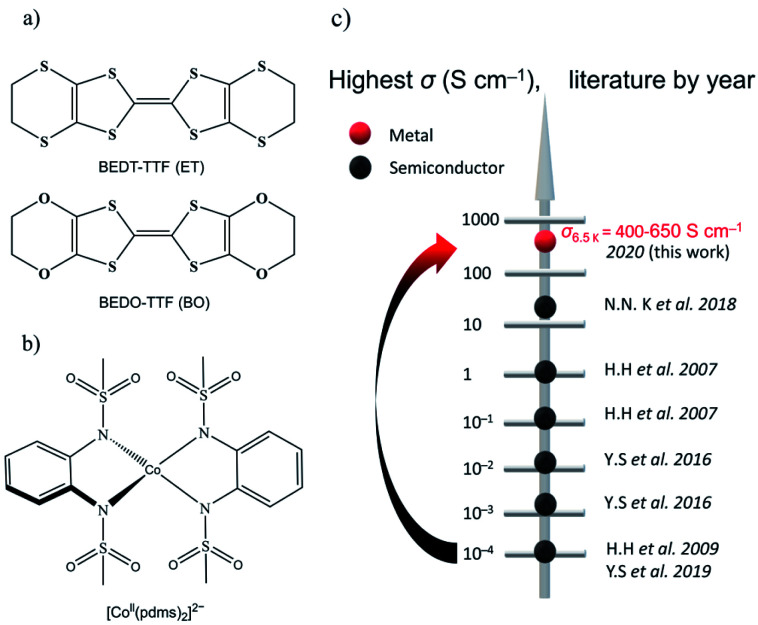
Organic conductors and single-molecule magnets. (a) Structural diagrams of BEDT-TTF (ET) and BEDO-TTF (BO). (b) Tetrahedrally coordinated structure of [Co(pdms)_2_]^2−^. (c) The evolution of the highest *σ* by year for the reported conducting SMM materials: black and red dots represent semiconductors and metals, respectively. The original literature abbreviated in this panel is listed in ref. 12.

The oxygen analogue of BEDT-TTF (ET) is BEDO-TTF (BO) (Fig. 1a), which is a well-known donor molecule to give metallic and superconducting ion radical salts.^14^ The difference between the two molecules lies in the fact that, in BO, the four sulfur atoms in the six-member rings of ET are substituted by smaller and lighter oxygen atoms in BO, which can effectively shorten the intermolecular S⋯S distance between the central TTFs. In crystals of metallic BO complexes, donor molecules aggregate into layered structures, forming uniform (such as I_3_-type) arrays connected by intrastack C–H⋯O hydrogen bonds and side-by-side heteroatom short contacts to form a stable 2D metal.^15^ Following this idea, (BEDO-TTF)_4_(ReF_6_)·3H_2_O was the first example of the application of BO molecules to preserve relatively high *σ* down to low temperature (*σ*_4.2 K_ = 47 S cm^−1^) for which the field-induced slow relaxation of magnetization was observed below 4 K, but no synergetic effects were reported.^16^ On the other hand, we selected a four-coordinate Co-based SMM, [Co(pdms)_2_]^2−^ (Fig. 1b). [Co(pdms)_2_]^2−^ is an outstanding SMM with a large negative zero-field splitting *D* value (*D* = −115 cm^−1^),^13^ which is necessary for high energy barrier SMMs. The low energy absorbance band at 1200 nm (calculated at 960 nm by the TD-CAM-B3LYP method) corresponds to the electron transition from the ^4^B_1_ orbital to the ^4^E orbital of the cobalt ion (Fig. S1†), indicating that the strong ligand field of the bis(sulfonamido) ligand in combination with a strong axial distortion accounts for the large *D* value. The first semiconducting SMM with *σ*_300 K_ = 0.22 S cm^−1^ was reported by H. Hiraga *et al.*^7*a*^ in 2007 and further explored by several groups in the following decade (Fig. 1c); the highest *σ* was reported to be 47 S cm^−1^ at 4.2 K by N. D. Kushch *et al.* in 2018,^16*a*^ but their SMMs were not metals. Here we report the first metallic conducting SMM material, β′′-(BO)_4_[Co(pdms)_2_]·3H_2_O, which has the highest *σ* value (400–650 S cm^−1^ at 6.5 K by measuring six single-crystals) in the family of conducting SMM materials and exhibits SMM behaviour in the antiferromagnetic phase with a *T*_B_ up to 11 K. The synergetic effect, which is expected from the band structure calculation, has been found as the antiferromagnetic interactions and magnetoresistance (MR) at low temperatures.

## Results and discussion

### Crystal structure

**Fig. 2:**
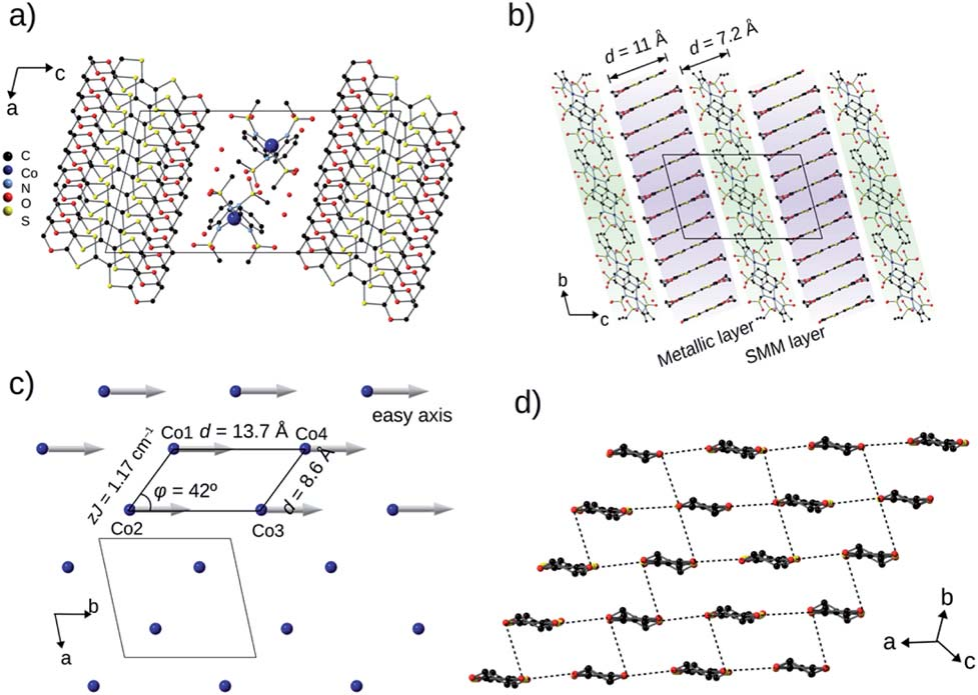
Crystal structure of **BO4** at 120 K. (a) A unit cell projected along the *b*-axis (black: C, blue: Co, pale blue: N, red: O and yellow: S). (b) Packing structure in the *bc* plane. The SMM layer (green background) alternates with the conducting layer (violet background) *via* short contacts, and the thicknesses of the SMM and conducting layers are 7.2 Å and 11.0 Å, respectively. (c) The arrangement of the Co ions in the SMM layer in the *ab* plane. The arrows represent the direction of the easy axis of the Co ions in the Co(pdms)_2_ molecules. The angle (*φ*) between three Co ions in one parallelogram is 42°, and the distances between two adjacent Co ions are 8.6 Å and 13.7 Å. (d) The BO molecules arranged side-by-side along the *a*-axis and face-to-face in the *a*–*b* direction. The black dotted lines represent the short contacts (S⋯S and S⋯O) between BO molecules along the *a*-axis and *a*–*b* direction. All hydrogen atoms are omitted for clarity.

**BO4** (CCDC: 1970380†) crystallized in the centrosymmetric triclinic *P*1̄ space group with four crystallographically independent BO molecules, one Co(pdms)_2_ unit and three water molecules (Fig. 2a) with unit cell dimensions *a* = 12.498(1) Å, *b* = 13.717(1) Å, *c* = 22.91(2) Å, *α* = 102.965(12)°, *β* = 101.501(14)° and *γ* = 99.116(18)° at 120 K. The total positive charge of the four BO molecules is compensated by [Co(pdms)_2_]^2−^ to give the charge neutrality of (BO)_4_[Co(pdms)_2_]·3H_2_O. Fig. 2b shows the packing structure in the *bc* plane. The SMM layer alternates with the metallic layer along the *c*-axis. The thicknesses of the layers are 7.2 Å and 11.0 Å, respectively, indicating that both of them are 2D nanosheets. Numbers of hydrogen-bonds (C–H⋯O) are observed between BO and SMM layers in addition to those within the BO layer, both of which stabilize the crystal structure (Fig. S4a and Table S2†). As shown in Fig. 2c, the Co ions are arranged to form an infinite arrangement of parallelograms in the *ab* plane, where the shortest distance between two Co ions is 8.6 Å, and the angle (*φ*) between three Co ions in one parallelogram is 42°. The magnetic easy axes of all Co(pdms)_2_ units are aligned along the *b*-axis to show a strong one-dimensional (1D) magnetic anisotropy (Fig. S5†). Fig. 2d shows the packing of the BO molecules in I_3_-type arrangement (corresponding to β′-type for ET salts^17^). While the intermolecular S⋯S and S⋯O atomic contacts shorter than the sum of van-der-Waals radii are observed along the *a*-axis (between side-by-side BO pairs) and along the *a*–*b* direction, significant magnitudes of intermolecular orbital overlap (transfer integral) are manifested along the *a*-axis and along the 3*a* + 2*b* direction (Fig. S4b†).

### Spectroscopic properties

In solid-state UV-Vis-NIR absorption spectra of **BO4**, several broad bands were observed at room temperature (Fig. S6a†). The lowest energy broad absorption extending to the near IR region (<7500 cm^−1^) corresponds to the intermolecular charge-transfer (CT) transition between partially oxidized BO molecules. The absorption band at around 12 000 cm^−1^ was assigned to the electronic transition within a BO radical cation (electronic transition from the second HOMO to HOMO).^15*b*^ In EPR spectra, a single peak with *g* = 2.004 was observed at room temperature (Fig. S6b†), confirming the presence of a BO radical. To further investigate the electronic structures, infrared reflectance spectroscopy was carried out on single crystals. Fig. S6c† shows room temperature reflectance spectra with the incident light polarized parallel to the *ab* plane. This reflectance spectrum was well fitted by a Drude–Lorentz model in the range of 550–7800 cm^−1^: plasma frequency (*ω*_ρ_), relaxation rate (*Γ*) and dielectric constant (*ε*_∞_) are evaluated as 6331.3 cm^−1^, 3394.2 cm^−1^, and 3.48, respectively. The reflectance spectrum indicates a highly conducting nature.^18^ Raman spectroscopy was carried out to determine the charge on the BO molecules to understand the degree of CT in the BO layer. The room-temperature Raman band at around 1480 cm^−1^ observed to be a superposition of two bands (∼1477 cm^−1^ and ∼1483 cm^−1^) was attributed to the totally symmetric C=C vibration of the BO (*v*_3_(A_g_) mode, Fig. S6d†). The position of the *v*_3_(A_g_) mode depends on the charge on BO and can be used to determine the charge density (*ρ*) using the following equation: *ρ* = (1524.9 − *ν*_3(obs)_ (cm^−1^))/109.0.^19^ The possibility of inhomogeneous charges on BO molecules is thus supposed; the calculated charges are +0.44 and +0.38, respectively.

### Magnetic properties

**Fig. 3:**
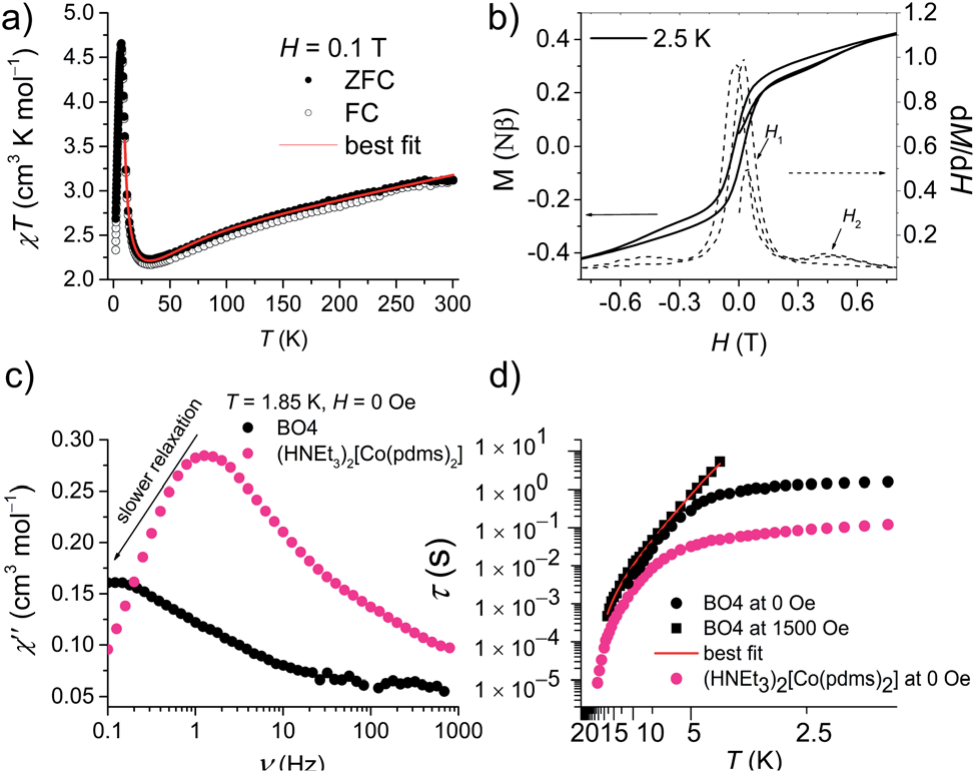
Magnetic properties. (a) Temperature dependence *χT* (black circles) in a 0.1 T field in field cooling (FC) and zero field cooling (ZFC) modes, respectively. The solid red lines represent the best fit using anisotropic spins. (b) Magnetic hysteresis of the magnetization at a sweep rate of 200 Oe·s^−1^ at 2.5 K and the first differential curve of d*M*/d*H* (dashed curves) in the range of −0.8 to +0.8 T. (c) A comparison of frequency dependences of out-of-phase *ac* magnetic susceptibilities *χ*′′ of **BO4** and (HNEt_3_)_2_[Co(pdms)_2_] in a field of 0 Oe. (d) *τ* as a function of *T* in fields of 0 and 1500 Oe for **BO4** and (HNEt_3_)_2_[Co(pdms)_2_]. The black circles, pink circles and the red curve represent the experimental data and best fit, respectively.

Fig. 3a shows the static magnetic susceptibility temperature products, *χT* of **BO4**, as a function of temperature in a 0.1 T field measured in zero field cooling (ZFC) and field cooling (FC) modes. The observed *χT* value of 3.22 cm^3^ K mol^−1^ at 300 K is 0.08 cm^3^ K mol^−1^ larger than the value expected for an *S* = 3/2 ion with *g* = 2.59 in (HNEt_3_)_2_[Co(pdms)_2_],^20^ which indicates that most of the paramagnetic susceptibility comes from Co(pdms)_2_. Hence, the contribution from the BO layer is estimated only to be around 2.7 × 10^−4^ cm^3^ mol^−1^, which is consistent with the metallic nature of the conducting layer. This *χ* value agrees well with the *χ*–*T* plot of (BO)_4_[Zn(pdms)_2_]·3H_2_O showing almost temperature-independent *χ* with the value of 2.8 × 10^−4^ cm^3^ mol^−1^ (Fig. S7†), which is comparable to *χ* at room temperature for ET based metallic salts.^21^ On cooling **BO4**, the *χT* value gradually decreased to 2.25 cm^3^ K mol^−1^ at 40 K due to the depopulation of Co ions. For *T* > 40 K, the Curie (*C*) and Weiss constants (*θ*) were estimated to be 1.93 cm^3^ K mol^−1^ and +10.2 K by using the Curie–Weiss law. Thus, the indirect ferromagnetic interactions below 10.2 K between Co ions mediated by the pdms ligand were assigned. A. A. Fraerman *et al.* have reported that the region with ferromagnetic long-range interaction of a magnetic dipole system in an orthorhombic lattice is stable.^22^ It has been shown that the ground state of the system depends on the rhombic angle (*φ*). If *φ* < 60°, the dipoles are ordered ferromagnetically and the *φ* value of 42° observed for **BO4** is regarded to be adequate to manifest ferromagnetic interactions in a parallelogram lattice (note, however, that the detailed mechanism behind magnetic interactions in this system is not obvious, and that direct magnetic–dipole interactions are expected to be weak here). After rising again, *χT* abruptly decreased upon cooling below 6.5 K to 2.68 and 2.33 cm^3^ K mol^−1^ in ZFC and FC modes, respectively, indicating that there are antiferromagnetic interactions. To model the static magnetic properties, the spin Hamiltonian 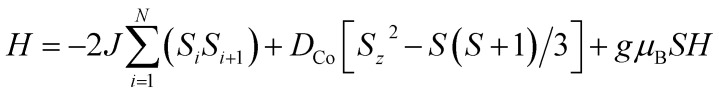, where *S*_i_ = *S*_Co_ = 3/2 and where *D*_Co_ stands for the zero-field splitting parameter for a Co^II^ ion, was employed. Parameters of *g*_(Co)_ = 2.19, *D*_Co_ = −67.3 cm^−1^, and *zJ* = 1.05 cm^−1^ under FC conditions and *g*_(Co)_ = 2.10, *D*_Co_ = −59.0 cm^−1^, and *zJ* = 1.17 cm^−1^ under ZFC conditions were obtained from fitting (eqn S1†). The small *zJ* values indicate that there are weak magnetic interactions between the Co(pdms)_2_ units in the SMM layer (Fig. 2c). The Néel temperature (*T*_N_), defined as peaks of the ln(*χT*)–*T*^−1^ plot, slightly shifted from 7.0 to 11 K when the magnetic field was increased from 200 to 10 000 Oe (Fig. S8†), indicating that there is antiferromagnetic ordering. The *χ* and *χT* values were investigated in fields of 10–1000 Oe below 20 K in ZFC and FC modes (Fig. S8†). The clear divergences of the *χ* and *χT* values in ZFC and FC modes when *T* < 11 K are direct evidence for blocking temperature (*T*_B_) of SMMs up to 11 K and *T*_N_ up to 6.5 K. To the best of our knowledge, this is the highest *T*_B_ among those reported for four-coordinate Co^2+^ based SMMs. Fig. 3b shows the field dependence of the magnetization (*M*–*H* curve) in the initial field sweep and the d*M*/d*H* curve measured at 2.5 K. A stepwise increase was observed, indicating the presence of the spin–flop transition in the field of *H*_1_ and *H*_2_ (*H*_2_ > *H*_1_). The spin–flop transitions were observed in an *H*_1_ of 430 Oe and an *H*_2_ of 4700 Oe at 2.5 K and an *H*_1_ of 480 Oe at 5 K (Fig. S9†), indicating a phase transition from an antiferromagnetic ground state to a paramagnetic ground state, consistent with the observation of a Néel peak in the *χ*–*T* plots in weak fields (Fig. S8†), and the resultant magnetic moments may come from the spin canting between localized Co spins and delocalized π spins. The spin–flop transition showed that the antiferromagnetic interactions were suppressed in an applied field of *H*_1_. *H*_1_ corresponds to the interactions between the SMM layers, which cause the second relaxation process observed in the alternating current (*ac*) magnetic susceptibility measurements (describe below). Therefore, the strength of antiferromagnetic interactions (*zJ*′) was calculated to be 0.0074 cm^−1^ by using a mean-field approximation model, 2|*zJ*′|*S*_T_^2^ = *gS*_T_*μ*_B_*H*_1_ with *S*_T_ = 3 and *g* = 2.0, where an *S*_T_ of 3 was the largest magnetic moment (two ferromagnetic Co(pdms)_2_ units) in the SMM layer. *H*_2_ could be assigned to the coupling between the SMM and BO layers. Magnetic hysteresis loops were observed up to 5 K with a remnant magnetization of 0.08 *N*β and a coherence field of 23 Oe at 2.5 K, indicating that **BO4** shows SMM behaviour (Fig. S10†).

Fig. 3c and S11† show the frequency dependence of the in-phase (*χ*′) and out-of-phase (*χ*′′) *ac* magnetic susceptibility, *χ*′′, at 0 Oe at various temperatures. Dual relaxation peaks in the range of 0.1–1000 Hz, with the first relaxation process at low frequency and the second relaxation process at high frequency. The first relaxation process corresponds to the reversal of the spins of the Co ions, and the second relaxation process is related to the antiferromagnetic interactions between two adjacent SMM layers. The second relaxation process was suppressed by an optimized 1500 Oe field (Fig. S12†). This magnetic field strength is strong enough to suppress the antiferromagnetic interactions (*H*_1_), leaving a single-relaxation process. Fig. 3d shows the temperature dependence of spin relaxation time (*τ*) extracted from *χ*′′ in fields of 0 Oe (Fig. S11†) and 1500 Oe (Fig. S12†). The *τ*for **BO4** below 11 K (Néel temperature) is much longer than that for (HNEt_3_)_2_[Co(pdms)_2_] at the 0 Oe field because of the ferromagnetic and antiferromagnetic interactions. Quantum tunnelling of magnetization (QTM) below 4 K was dominant, but it became suppressed at a frequency lower than that for the starting material due to the presence of weak antiferromagnetic interactions (*H*_1_). The energy barrier (*U*_eff_) was determined by using the relaxation process at an optimized magnetic field. By considering the direct, Raman and Orbach processes for the fitting give no reasonable results. Thus, we consider here Orbach and Raman processes by using eqn (1).1
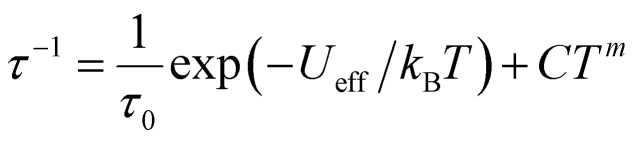
*τ* is best fitted using the following parameters: *C* = 1.42 × 10^−5^ s^−1^ K^−*m*^, *m* = 6.37, *τ*_0_ = 2.34 × 10^−4^ s, and *U*_eff_ = 29.21 cm^−1^. *Τ*_0_ for **BO4** is almost six orders of magnitude lower than that for (HNEt_3_)_2_[Co(pdms)_2_] (*τ*_0_ = 1.31 × 10^−10^ s).^20^ Although the magnetic properties of **BO4** are completely different from those of the starting material and *U*_eff_ is smaller than that of (HNEt_3_)_2_[Co(pdms)_2_], *U*_eff_ is similar to that of our recently reported conducting SMM (TTF)_2_[Co(pdms)_2_] (*C* = 2.35 × 10^−5^ s^−1^ K^−*m*^, *m* = 6.59, *τ*_0_ = 2.08 × 10^−4^ s, *U*_eff_ = 24.1 cm^−1^).^13^ One possibility is that the *D* value is −67 cm^−1^, whereas for (HNEt_3_)_2_[Co(pdms)_2_], it is −115 cm^−1^.^20^

### Electrical conductivity and MR effect

**Fig. 4:**
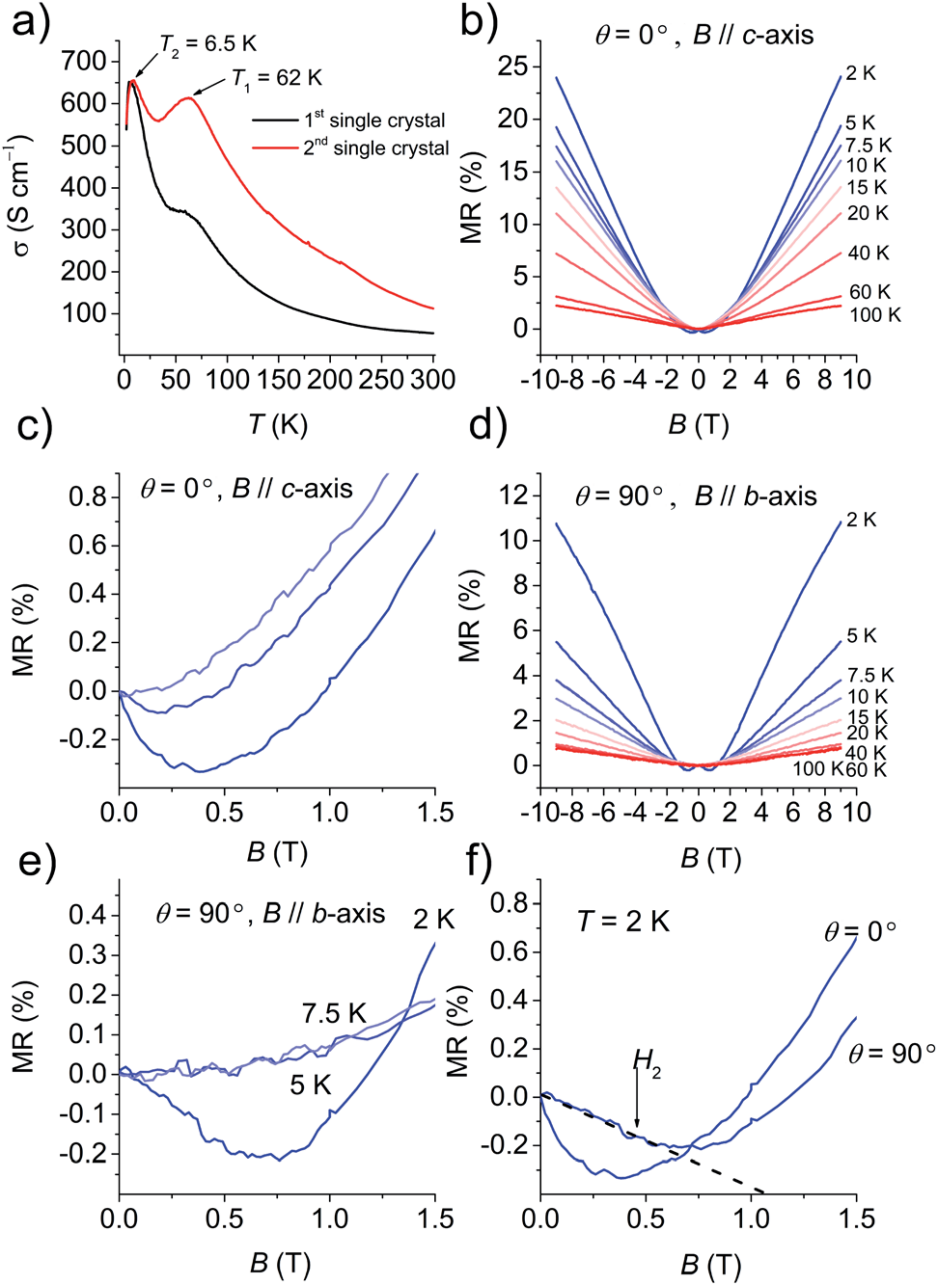
Electrical conductivity and magnetoresistance effects. (a) Temperature dependence of *σ* of two single-crystals using a standard four-probe technique. (b) Magnetic field dependence of MR at different temperatures with the magnetic field perpendicular to the 2D plane, *θ* = 0°, *B*‖*c*-axis. (c) MR–*B* plot in the range of 0–1.5 T at 2 K, 5 K and 7.5 K at *θ* = 0°. (d) Magnetic field dependence of MR at different temperatures with the magnetic field parallel to the 2D plane, *θ* = 90°, *B*‖*b*-axis. (e) MR–*B* plot in the range of 0–1.5 T at 2, 5 and 7.5 K with *θ* = 90°. (f) The MR–*B* plot in the range of 0–1.5 T at 2 K with *θ* = 0° and 90°.

Fig. 4a and S13† show the temperature dependence of *σ* at various pressures based on six single-crystals. At ambient pressure, *σ* (=50–110 S cm^−1^ at 295 K) increased with a decrease in temperature, behaving as a metal above 62 K. Then *σ* decreased down to 32 K, then increased again up to *σ* = 400–650 S cm^−1^ at 6.5 K, and finally drastically decreased to *σ* = 560 S cm^−1^ at 2 K. The change ratio *σ*_(2 K)_/*σ*_(300 K)_ was 5–10. The decrease in *σ* at ∼62 K was completely suppressed by coating the crystal with Apiezon N grease, which is known to induce a weak pressure effect of ∼0.03 GPa on the crystal. However, the decrease in *σ* at low temperature could not be suppressed even when pressures up to 2.0 GPa were applied. To investigate the origin of the anomaly at low temperature under *σ*, the magnetic field was swept between −9 T and 9 T. The magnetic field *B* was oriented perpendicular to the current direction with an angle *θ* between the magnetic field and conducting plane (*ab* plane) adjusted by the rotator probe (Fig. S14a and S14b†). MR is defined by the equation: MR (%) = [*ρ*(*B*,*T*) − *ρ*(0,*T*)]/*ρ*(0,*T*) × 100%, where *ρ* is the resistivity. Fig. 4b shows the MR effect under *θ* = 0° in the range of 2–100 K with symmetrical evolutions with respect to the field direction. The linear positive MR with 24% resistance increases at 2 K, and that with 2% resistance increases at 100 K in the field of 9 T. A negative MR was observed below 7.5 K in the low magnetic field region (Fig. 4c). In order to clarify whether the negative MR (*θ* = 0°) was caused by weak 2D localization or the interactions with the magnetic layer, the angle dependence of MR was examined. Fig. 4d shows the MR effect at *θ* = 90° in the range of 2–100 K. The linear positive MR with 11% resistance increases at 2 K and that with 0.7% resistance increased at 100 K under a 9 T field. Furthermore, a negative MR was observed below 5 K in the low magnetic field region (Fig. 4e). The negative MR was observed both perpendicular and parallel to the current at 2 K (Fig. 4f). A parallel magnetic field (*θ* = 90°) should have no effect on 2D localized electrons,^23^ however, a negative MR was also observed at 90° in contrast to the 2D localization model. This fact indicates that the decrease in *σ* observed below 6.5 K cannot be attributed to 2D weak localization. There must be interactions between the conducting and SMM layers in this temperature range.

### Electronic structure calculations

**Fig. 5:**
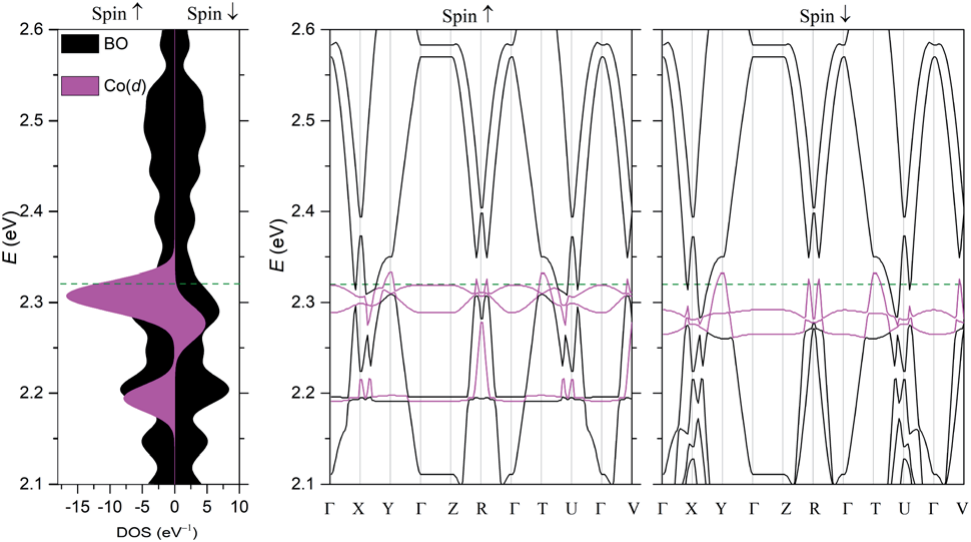
DFT calculations. Electronic band structure based on the crystal structure determined by using DFT calculations. High symmetry points in the crystal reciprocal lattice: *Γ* = (0, 0, 0), *X* = (0.5, 0, 0), *Y* = (0, 0.5, 0), *Z* = (0, 0, 0.5), *R* = (0.5, −0.5, 0.5), *T* = (0, 0.5, −0.5), *U* = (0.5, 0, −0.5), and *V* = (0.5, −0.5, 0). The Fermi energy level (green dashed line) is set at 2.32 eV. The magenta and black areas and curves indicate Co and BO electrons, respectively.

**Fig. 6:**
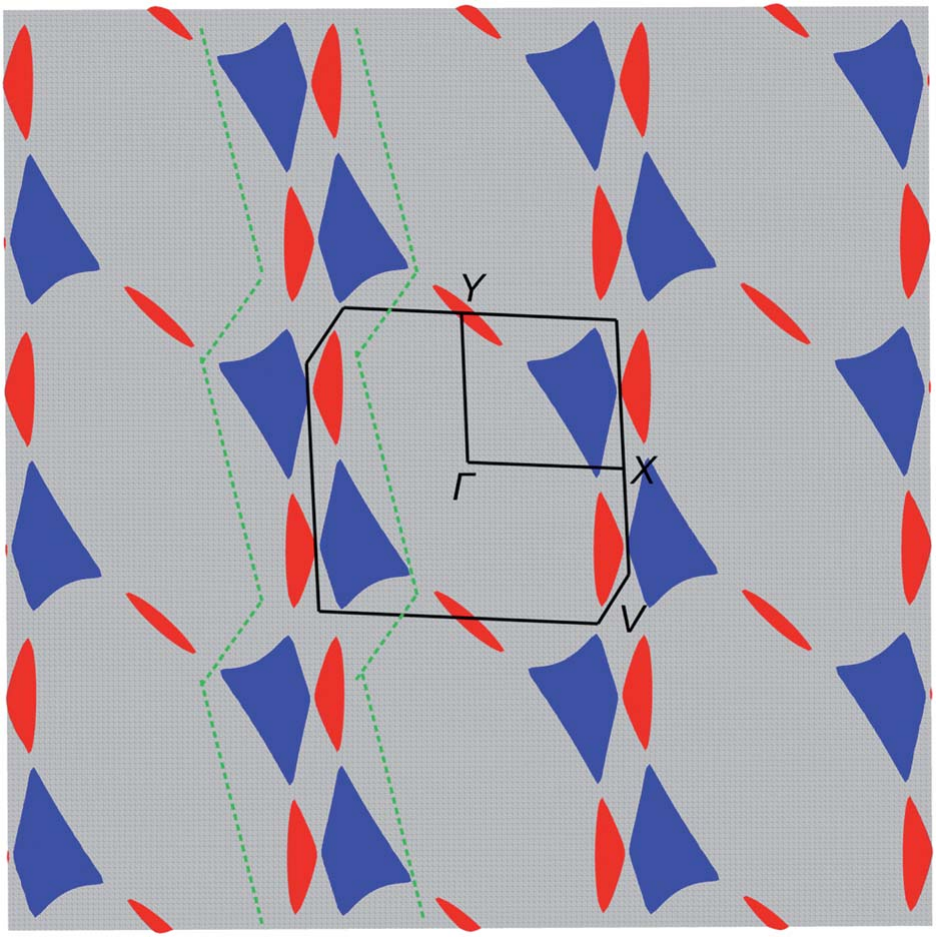
DFT calculations. Fermi surface of **BO4** in an extended Brillouin zone, which is composed of electron (blue) and hole (red) pockets associated with the (*Γ*, *X* and *Y*) symmetry points in the bands. Black lines represent the first Brillouin zone, and green dotted lines represent the hidden 1D Fermi surface.

The electronic structure was calculated using DFT (Fig. 5) and extended Hückel tight-binding (EHTB) methods (Fig. S15†). The calculated results from the two methods agree with each other at least qualitatively. Fig. 5 shows the results from DFT-based density of state (DOS) and band structure calculations. The DOS has a small amount of BO electron density at the Fermi level, indicating that the BO layer has semimetallic characteristics. The total bandwidth is calculated to be 0.78 eV, which is close to that of the organic superconductor (BEDO-TTF)_2_ReO_4_·H_2_O.^24^ The bands of **BO4** near the Fermi level mainly consist of eight HOMOs of the BO units, which are largely dispersed due to the effective HOMO overlap of the BO pairs in the “side” and “diagonal” directions (Fig. S16†). The undistinguished energy difference between the bands with up and down spin indicates the paramagnetic characteristic of the BO layer. The flat HOMO and HOMO-1 bands of the two Co units were mixed with the BO bands, causing hybridization between them. Thus, the angular momentum of the Co(3d) electrons could directly mix into that of the carrier (itinerant BO electrons) despite the small contribution of the Co(3d) orbitals to the HOMO. The Fermi level slightly cuts the conduction bands in *ΓXY* and *ΓTU* planes in the reciprocal lattice (high symmetry points are defined in Fig. S17†). There is almost no dispersion along the *ΓZ* direction, indicating that **BO4** is a 2D conductor. In addition, near the symmetry points *R* and *V*, the valence and conduction bands take the shape of the upper and lower halves of a conical surface with tiny energy gaps, behaving as a semimetallic electronic structure (Fig. 5 and Fig. S18†). The Fermi surfaces, which are composed of two independent triangular-like hole tubes (Fig. S19†) and one unique quadrangular-like electron tube (Fig. S20†) and the symmetry-operation derived ones in the first Brillouin zone are shown in Fig. 6. This is because the intermolecular interaction is two-dimensional due to the I_3_-type BO packing, and the Fermi surfaces are arranged to give the distorted quadrilateral-like and triangular-like islands along the *a*–*b* direction due to the shape and size of the Brillouin zone. All electron and hole tubes are at the edge of the first Brillouin zone with one dependent hole tube along the *ΓY* direction, and the other hole tubes are aligned in the *ΓV* direction close to the electron tube without crossing. Thus, the results of the calculations indicate that **BO4** is a 2D semimetal.

### Discussion

The most striking physical property of **BO4** is the simultaneous manifestation of SMM nature and metallic *σ*. While *σ* shows semiconducting behaviour from 62 to 32 K and below 6.5 K, the temperature region for the metallic conduction includes the one for SMM behaviour (*T*_B_ = 11 K). The Co(pdms)_2_ molecules are arranged in an ordered 2D narrow sheet (*d* = 7.2 Å), which causes the magnetic anisotropy of each Co(pdms)_2_ unit to be nearly aligned with the *b*-axis, affording a strong magnetic anisotropy. The ferromagnetic ordering observed between 10.2 K and 6.5 K is caused by weak intermolecular interactions between the Co(pdms)_2_ molecules due to the small rhombic angle in the magnetic layer. Subsequently, antiferromagnetic ordering was observed below 6.5 K assigned to the weak interactions between the SMM and conducting layers (d–π interactions). Thus, the weak inter- and intralayer interactions and the strong magnetic anisotropy maintain the SMM behaviour in a region of antiferromagnetic ordering. In other words, the Co(pdms)_2_ molecules are not significantly influenced by the surrounding Co ions, which essentially protects the SMM behaviour. On the other hand, we found that the *T*_B_ of the Co(pdms)_2_ units significantly increased and the magnetic relaxation times increased below *T*_N_, which are caused by the interactions between conducting electrons and the SMM.

For electron transport, the first decrease in *σ* at 62 K was suppressed by applying very low pressure (*P* = 0.03 GPa), which has been reported for several low dimensional organic metals,^25^ such as (BEDO-TTF)_2_ReO_4_·H_2_O and (TMTSF)_2_NO_3_, with a metal–insulator transition into a charge density wave (CDW) or spin density wave (SDW) state at low temperature.^26^ The *σ* value of (BEDO-TTF)_2_ReO_4_·H_2_O decreases several times due to the electronic instability involving the nesting of the hidden 1D Fermi surfaces (formed by the 2D hole and electron pockets).^27^**BO4** has a similar arrangement of 2D Fermi surfaces of hole and electrons, and the decrease in *σ* at 62 K is ascribed to the formation of a CDW state due to the hidden 1D Fermi surface. The nesting of the 1D hidden Fermi surface along the *ΓY* direction (green dotted lines in Fig. 6) can effectively shrink a part of the Fermi surface in β′′-type 2D organic semimetals.^28^ However, some of the remaining Fermi surfaces continue to exhibit metallic behaviour at low temperature, as is the case for **BO4** below 32 K. The conductivity in a 2D semimetal with 1D hidden nesting is very sensitive to the pressure, and even very weak pressure (∼0.03 GPa) is enough to induce a change in *σ*.^29^

The second decrease in *σ* below 6.5 K is insensitive to pressure. In this temperature range, a negative MR was observed for both perpendicular and parallel fields with respect to the conducting layer. Thus, the d–π interactions between the SMM and BO layers are important. Negative MRs were observed in fields perpendicular and parallel to the conducting layer up to ∼0.5 T, which is consistent with the characteristic spin–flop field (*H*_2_) in the MH curve. However, we should observe the corresponding distinct anomaly in the negative MR at the spin–flop transition (*H*_2_),^30^ and the absence of *H*_2_ in the MR measurement could be explained by the superposition of the largely positive MR enhanced by the peculiar semimetal-like electronic structure.

It is well known that a highly correlated electron system tends to adopt an antiferromagnetic insulating state.^31^ For the SMM layers, the *T*_B_ of Co(pdms)_2_ was observed up to 11 K because of ferromagnetic coupling below 10.2 K. The increase in *T*_B_ originates from the intrinsic ferromagnetic interactions. On the other hand, for the BO layers, the *ab* plane is metallic, and some electron density remains, although the partial nesting of the Fermi surface could occur. Thus, there is a possibility that the BO electrons are antiferromagnetically coupled with the SMM electrons along the *b*-axis below 6.5 K. Due to the two-dimensional conducting nature and strong magnetic anisotropy of the Co(d) electrons near the Fermi surfaces, synergetic effects could be observed. Furthermore, the presence of synergetic effects was supported by the low-temperature MR measurements where significant negative MR effects were observed in perpendicular and parallel directions.

## Conclusions

Slow electrocrystallization of [Co(pdms)_2_]^2−^ and neutral BEDO-TTF afforded β′-(BEDO-TTF)_4_ [Co(pdms)_2_]·3H_2_O (**BO4**), which showed the simultaneous manifestation of metallic conduction and SMM behaviours for the first time. Antiferromagnetic ordering mediated by π-electrons with SMMs was observed for the first time. By analysing the low-temperature magnetic phase transition and angle-dependence of magnetotransport, we found that the 2D conducting plane and strongly magnetic anisotropy played key roles in the synergetic effects in this layered material. From this aspect, SMMs have big advantages in molecule-based quantum spintronics compared to bulk magnets due to not only their high magnetic densities but also their strong magnetic anisotropies in SMMs. By combining them with high dimensional molecular conductors, such as two-dimensional BO and ET packing motifs, it is possible to observe such synergetic effects. Thus, from our studies on **BO4**, more stable metallic SMMs can be designed with the hope of producing superconducting SMM materials in the near future.

## Conflicts of interest

There are no conflicts to declare.

## Supplementary Material

SC-011-D0SC04040A-s001

SC-011-D0SC04040A-s002
