# Supplementary material for: Simultaneous manifestation of metallic conductivity and single-molecule magnetism in a layered molecule-based compound
Source: Chem Sci. 2020 Sep 1;11(41):11154–61. doi: 10.1039/d0sc04040a (PMC8162363; doi:10.1039/d0sc04040a)
Supplement: SC-011-D0SC04040A-s001 [file SC-011-D0SC04040A-s001.pdf]

Electronic Supplementary Material (ESI) for Chemical Science.
This journal is © The Royal Society of Chemistry 2020

Supporting Information for

Simultaneous Manifestations of Metallic Conductivity and Single-Molecule Magnetism in a Layered Molecule-based Compound

Yongbing Shen, [*,a] Hiroshi Ito, [*,b] Haitao Zhang, [c] Hideki Yamochi, [d,e] Seiu Katagiri, [b] Shinji K. Yoshina, [b] Akihiro Otsuka, [d,e] Manabu Ishikawa, [d] Goulven Cosquer, [f] Kaiji Uchida, [a] Carmen Herrmann, [c] Takefumi Yoshida, [a] Brian K. Breedlove, [a] and Masahiro Yamashita, [*,g,a]

[a] *Department of Chemistry, Graduate School of Science, Tohoku University, Sendai, Japan Address here.*
[b] *Department of Applied Physics, Nagoya University, Chikusa-ku, Nagoya 464-603, Japan.*
[c] *Institute of Inorganic and Applied Chemistry, University of Hamburg, Martin-Luther-King-Platz 6, 20146 Hamburg, Germany.*
[d] *Division of Chemistry, Graduate School of Science, Kyoto University, Sakyo-ku, Kyoto 606, 8502, Japan.*
[e] *Research Center for Low Temperature and Materials Sciences, Kyoto University, Sakyo-ku, Kyoto 606-8501, Japan.*
[f] *Research Group of Solid Material Chemistry, Graduate School of Science, Hiroshima University, 1-3-1 Kagamiyama, Higashihiroshima, Hiroshima, 739-8526, Japan.*
[g] *School of Materials Science and Engineering, Nankai University, Tianjin 300350, China.*

Table of Contents

Experimental details. ……………………………………………………………4-5

Figure S1. Measured (magenta) and calculated (blue) solid-state UV-Vis-NIR spectrum of $(HNEt_3)_2[Co(pdms)_2]$……………………………………………………....6

Figure S2. Typical size of single-crystal of **BO4**…………………………………………..7

Figure S3. The powder X-ray patterns of **BO4**……………………………………………7

Figure S4. a) The C-H···O type hydrogen-bonds between metallic and SMM layers. b) the direction of *a, b* and 3*a*+2*b* in BO layer………………………………………..8

Figure S5. The magnetic anisotropy energy calculation of **BO4**…………………………...9

Figure S6. Spectroscopic properties of **BO4**……………………………………10

Figure S7. The static magnetic susceptibility of $(BO_4)[Zn(pdms)_2]\cdot 3H_2O$……………...11

Figure S8. Magnetic susceptibility, χ and magnetic susceptibility temperature product, χT as a function of temperature at 10, 100, 200, 500 and 1000 Oe field in field cooling (FC) and zero field cooling (ZFC) modes……………………………..12

Figure S9. The field dependence of magnetization (M-H curves) in the initial field sweep and its d*M*/d*H* curves measured at 2.5, 3, 4 and 5 K………………….13

Figure S10. The M-H curves at 2.5, 3, 4 and 5 K, a) M-H in the magnetic field of –5–+5 T b) Enlarged view of M-H curve in the short magnetic range of –1–+1 T….14

Figure S11. Frequency dependence of in-phase (χ') and out-of-phase magnetic susceptibility (χ'') at 0 Oe…………………………………………………15

Figure S12. Frequency dependence of in-phase (χ') and out-of-phase magnetic susceptibility (χ'') at different magnetic field………………………………..16

Figure S13. Pressure effects on the electrical conductivity as a function of temperature…………………………………………………………………...17

Figure S14. a) Four-probe technique for electrical conductivity measurement with typical size of single crystals. b) The angle determination by adjusting the orientation of the magnetic field and electrical current……………………………………18

Figure S15. The band structure calculated by extended Hückel method………………19

Figure S16. Three different short contacts between adjacent BO units…………………20

Figure S17. High symmetry points for the first principle band structure calculation…….20

Figure S18. The DFT-based band energy dispersion of **BO4** in Co spin up and down states……………………………………………………………………………..………21

Figure S19. The arrangement of hole tubes in the extended first Brillouin zone by DFT calculation……………………………………………………………………….….22

Figure S20. The arrangement of electron tubes in the extended first Brillouin zone by DFT calculation……………………………………………………………………..……23

Table S1. Hückel parameters for the band calculation based on the extended Hückel method………………………………………………………………………………..24

Table S2. Summary of the hydrogen bonds (C-H···O) between layers………………….24

Equation S1……..………………………………………………………………….…..……24

References for SI……………………………………………………………..……..25

Experimental details

H_2pdms, $CoCl_2$, and organic solvents were commercially purchased and used without any further purification. Neutral BO was obtained as the by-product in the synthesis of ethylenedioxytetrathiafulvalene[1] and purified by recrystallization from cyclohexane. $(HNEt_3)_2[Co(pdms)_2]$ was synthesized following reported procedures.[2]

Preparation of β"-$(BO)_4[Co(pdms)_2]\cdot 3H_2O$: Neutral BO (15 mg) and $(HNEt_3)_2[Co(pdms)_2]$ (100 mg) were dissolved in a mixture of 1,1,2-trichloroethane (18 mL), ethanol (1 mL) and two drops of distilled water, and the resulting solution was stirred for 30 min and then filtered. Then the filtrate was placed in an H-shaped cell under N_2 gas atmosphere. The dc current (0.5 μA) was then applied. Black thin plate-like crystals with typical size of 0.8 × 0.2 × 0.02 mm^3 (Fig. S2) grew on the anode over 15 days. The crystals were collected, washed with a small amount of ethanol and dried in air. Yield: ~11.1% (based on BO). Elemental analysis: $C_{56}H_{58}N_4O_{27}S_{20}Co_1$ cal.: C, 35.04; H, 3.05; N, 2.92, S, 33.41. Found: C, 35.23; H, 3.32; N, 2.98; S, 33.46. The phase purity was confirmed by using powder X-ray diffraction (Fig. S3).

Physical characterization: Single-crystal crystallographic data were collected at 120 K on a Rigaku Saturn70 CCD Diffractometer (Rigaku, Tokyo, Japan) with graphite-monochromated Mo Kα radiation (λ = 0.71073 Å) produced by a VariMax microfocus X-ray rotating anode source. Data processing was performed using the Crystal-Clear crystallographic software package.[3] The structures were solved by using direct methods included in SIR-92,[4] and refinement was carried out using SHELXL-2013.[5] The non-H atoms were refined anisotropically using weighted full-matrix least squares, and H atoms attached to the C atoms were positioned using idealized geometries and refined using a riding model. Elemental analysis was performed at the Research and Analytical Centre for Giant Molecules, Tohoku University.

UV-Vis spectra were acquired in the solid-state as KBr pellets on a Shimadzu UV-3100pc (Shimadzu, Kyoto, Japan). Reflectance IR spectra were acquired on a JASCO IRT-5000 microscope and an FT-IR-6200YMS infrared spectrometer (JASCO, Tokyo, Japan) in the *ab* plane of a single crystal. IR spectra were acquired in the solid state as KBr pellets on a FT-IR-6200YMS infrared spectrometer (JASCO, Tokyo, Japan). Raman spectroscopy was performed on single crystals using a Micro Laser Raman Spectrometer LabRam H-800 at an excitation of 532 nm. EPR: The EPR spectrum was acquired at room temperature by using a JEOL JES-FA100.

Magnetic susceptibility measurements were conducted on polycrystalline samples using a Quantum Design SQUID magnetometer MPMS-7L. Diamagnetic corrections were estimated from Pascal's constants (-960×10^{-6} cm^3 mol^{-1}).[6] Ac measurements were performed in the frequency range of 0.1–1000 Hz. Temperature dependences of σ and MR of single crystals were determined with a four-probe method using a Quantum Design PPMS 6000 equipped with horizontal rotator probe.

Electronic structure calculations: The band structure was calculated by using periodic boundary conditions (PBC) Kohn-Sham density functional theory (DFT) calculations with the VASP[7] package employing the Perdew–Burke–Ernzerhof (PBE) exchange-correlation functional.[8] PAW pseudopotentials and plane-wave basis sets with cutoff energies of 800 eV were used. A 4 × 4 × 2 Monkhorst-Pack *k*-mesh was employed for SMM and metallic layers, and a 4 × 4 × 1 Monkhorst-Pack *k*-mesh was employed only for the metallic layer. In addition, the band structure was calculated using an extended Hückel method in the software package developed by T. Mori *et al.*[9] via a the tight-binding band approximation in which transfer integrals were evaluated with $t = ES$, where E is the energy of the HOMO (= –10 eV) and S is the overlap integrals between the HOMOs of the different molecules. The Hückel parameters used are listed in Table S1.

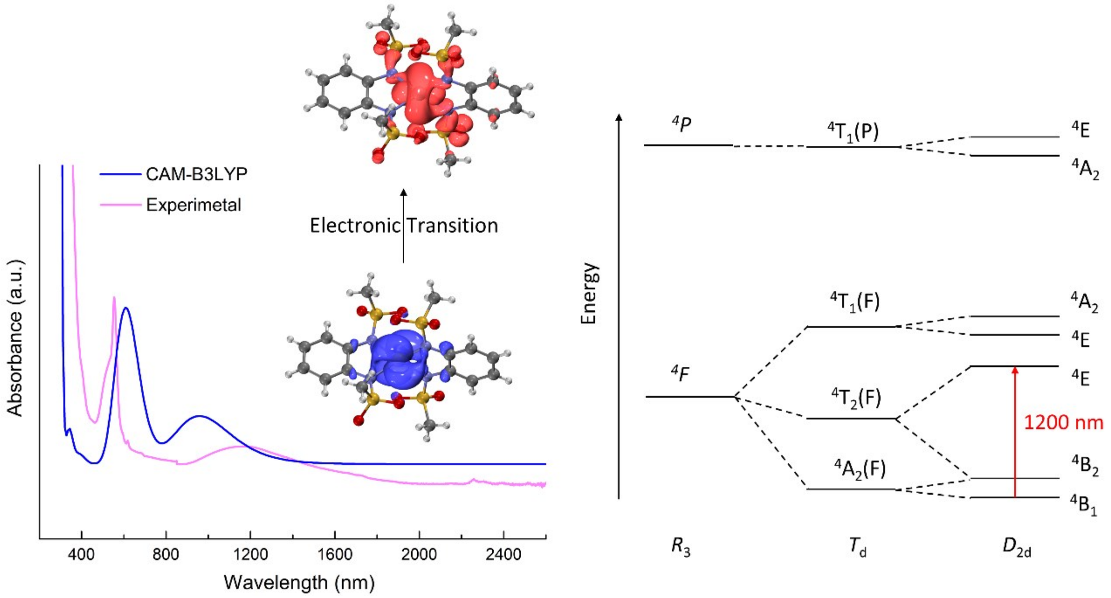

Figure S1. Measured (magenta) and calculated (blue) solid-state UV-Vis-NIR spectrum of $(HNEt_3)_2[Co(pdms)_2]$. The absorbance at 1200 nm, which was calculated by CAM-B3LYP at 960 nm, mainly corresponds to the d-d electron transition on Cobalt ion. Energy level diagram showing the splitting of the free cobalt ion quartet states under the influence of a ligand field with T_d and D_{2d} symmetries.[2]

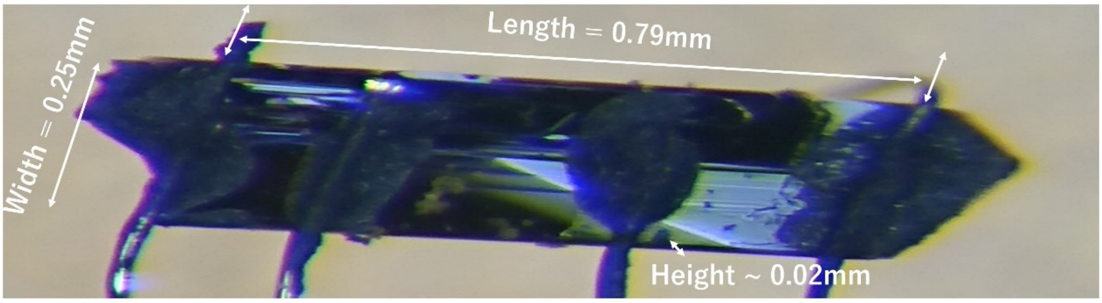

Figure S2. A typical size of single-crystal of **BO4** with a dimension of length = 0.79 mm, width = 0.25 mm and thickness = ~0.02 mm. Four Pt electrodes are attached with carbon paste for conductivity measurements.

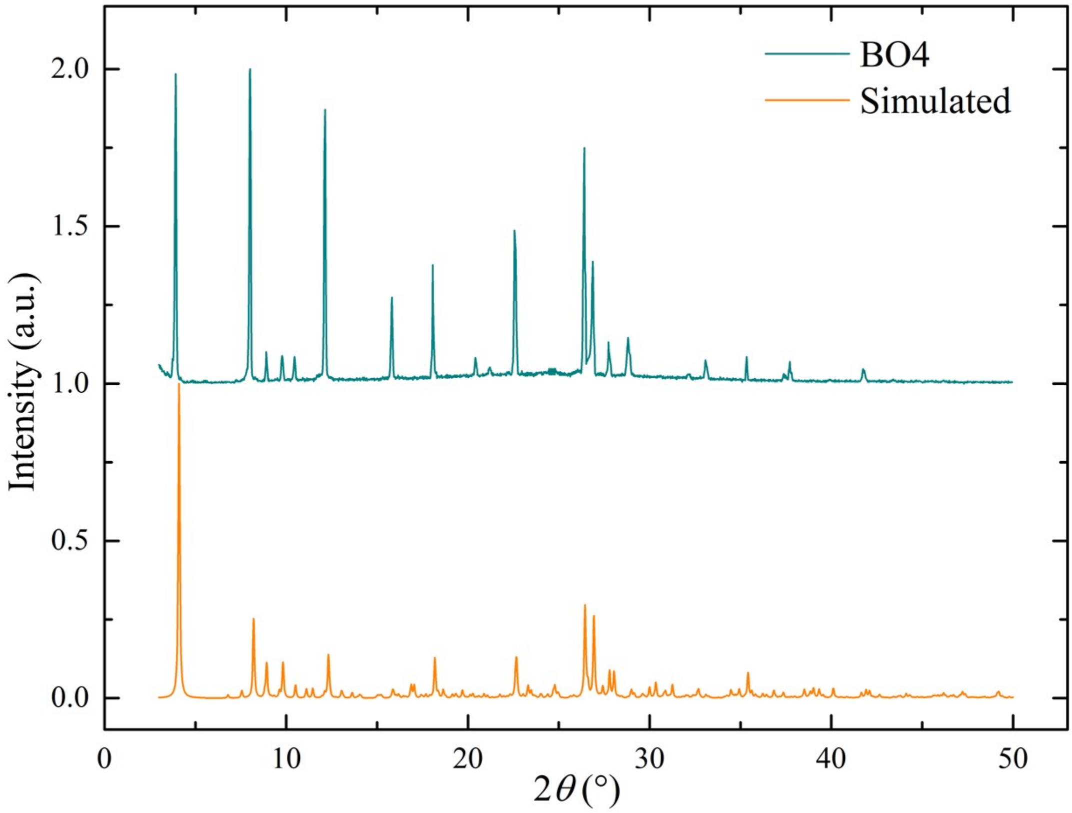

Figure S3. The powder X-ray patterns of **BO4** recorded at room temperature (dark cyan) and simulated based on the single-crystal data at 120 K (orange).

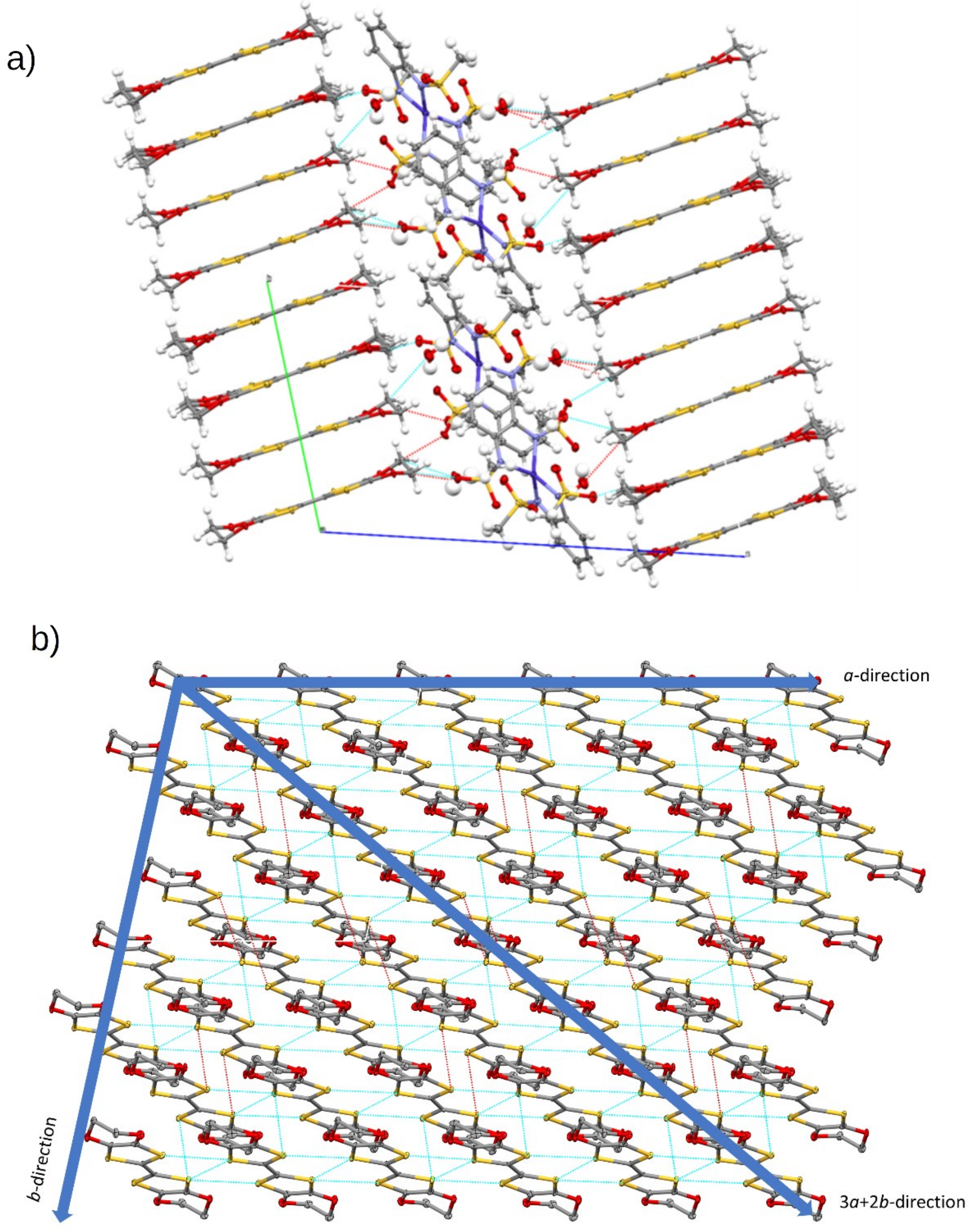

Figure S4. a) The C-H⋯O type hydrogen-bonds between metallic and SMM layers (dotted red and cyan lines). b) the direction of *a, b* and 3*a*+2*b* in BO layer.

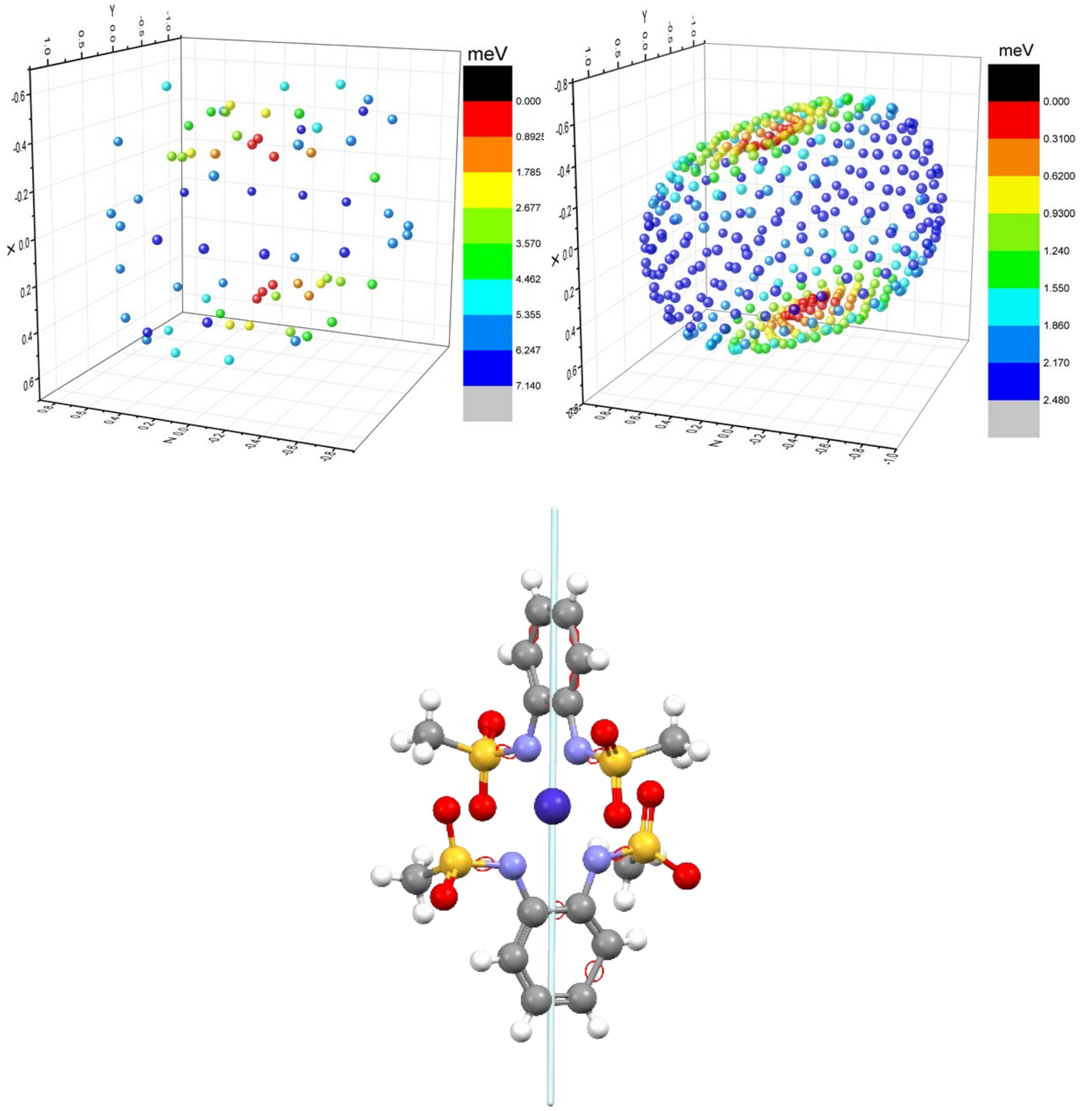

Figure S5. The magnetic anisotropy energy of β"-$(BO)_4[Co(pdms)_2]\cdot 3H_2O$ (left) and $[Co(pdms)_2]^{2-}$ (right).The magnetic anisotropic energies were calculated taking in account spin-orbit coupling, employing the zeroth order regular approximation (ZORA) as implemented in VASP. The easy axis of $[Co(pdms)_2]^{2-}$ in a supercell is along a direction of (-0.0992176964, 0.9371809859, -0.3344363145; Cartesian) (-0.04356a, 1b, -0.24708c; fractional), which almost overlaps with the principal axis of the molecule and also agrees with the previous reports.[2]

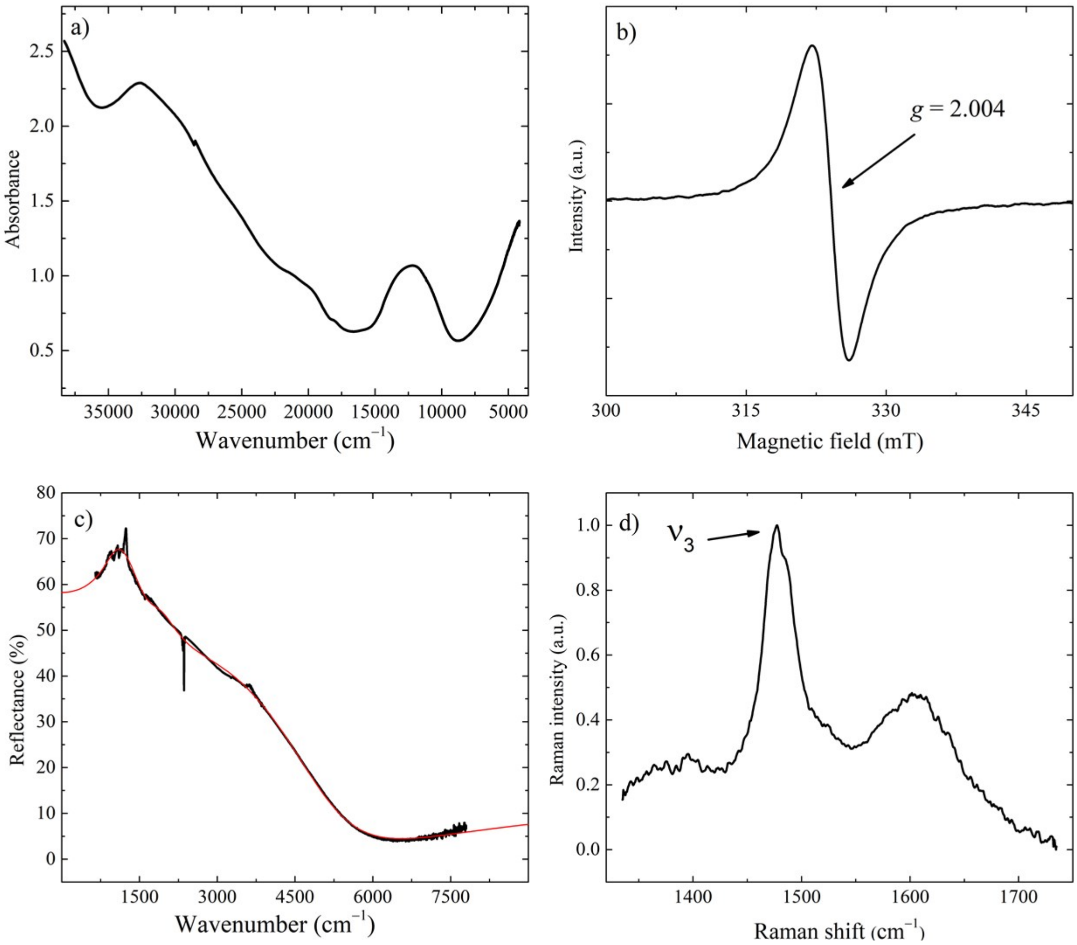

Figure S6. a) The solid-state UV-Vis-NIR absorption spectrum in the range of 4000-37500 cm^{-1} at room temperature with KBr as the background. b) EPR signal was recorded on polycrystals at room temperature. c) Room temperature reflectance spectrum with the incident light polarized parallel to *ab* plane. The red curve represents the best fit by Drude-Lorentz fitting model. d) Raman spectrum measured on the single crystals with an excitation of 532 nm at room temperature.

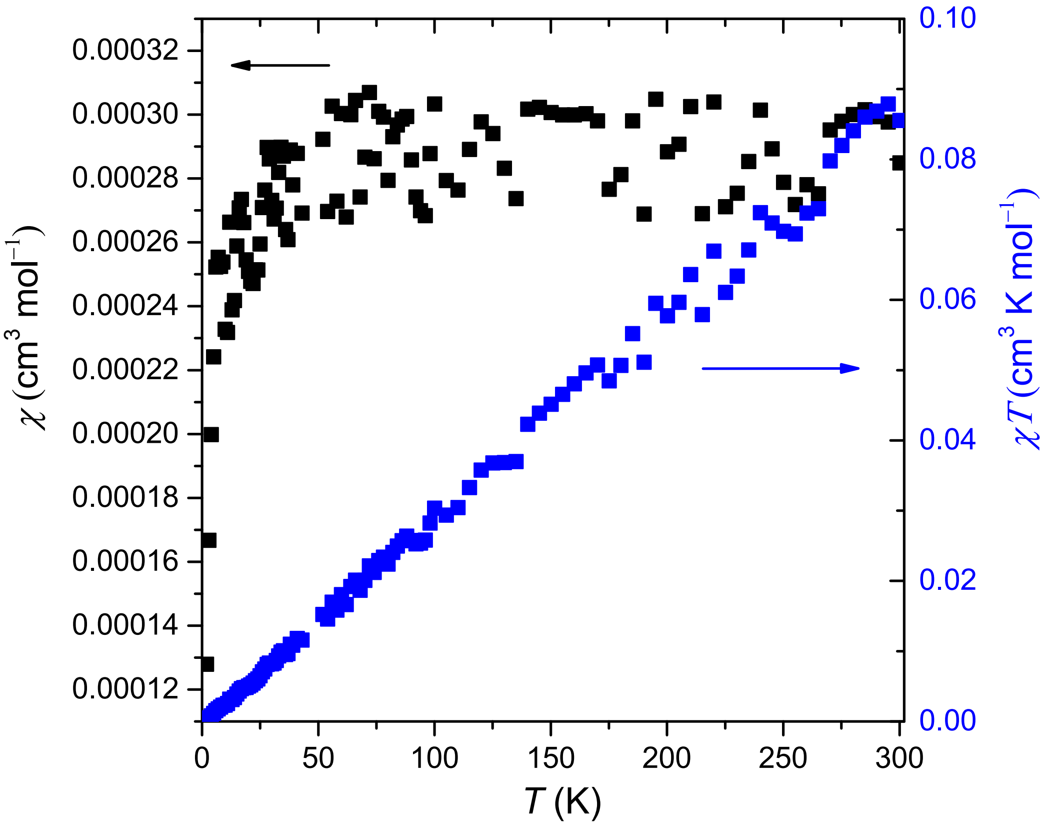

Figure S7. The static magnetic susceptibility χ (black) and the static magnetic susceptibility temperature χT (blue) as a function of temperature at 1000 Oe field for $(BO)_4[Zn(pdms)_2]\cdot 3H_2O$.

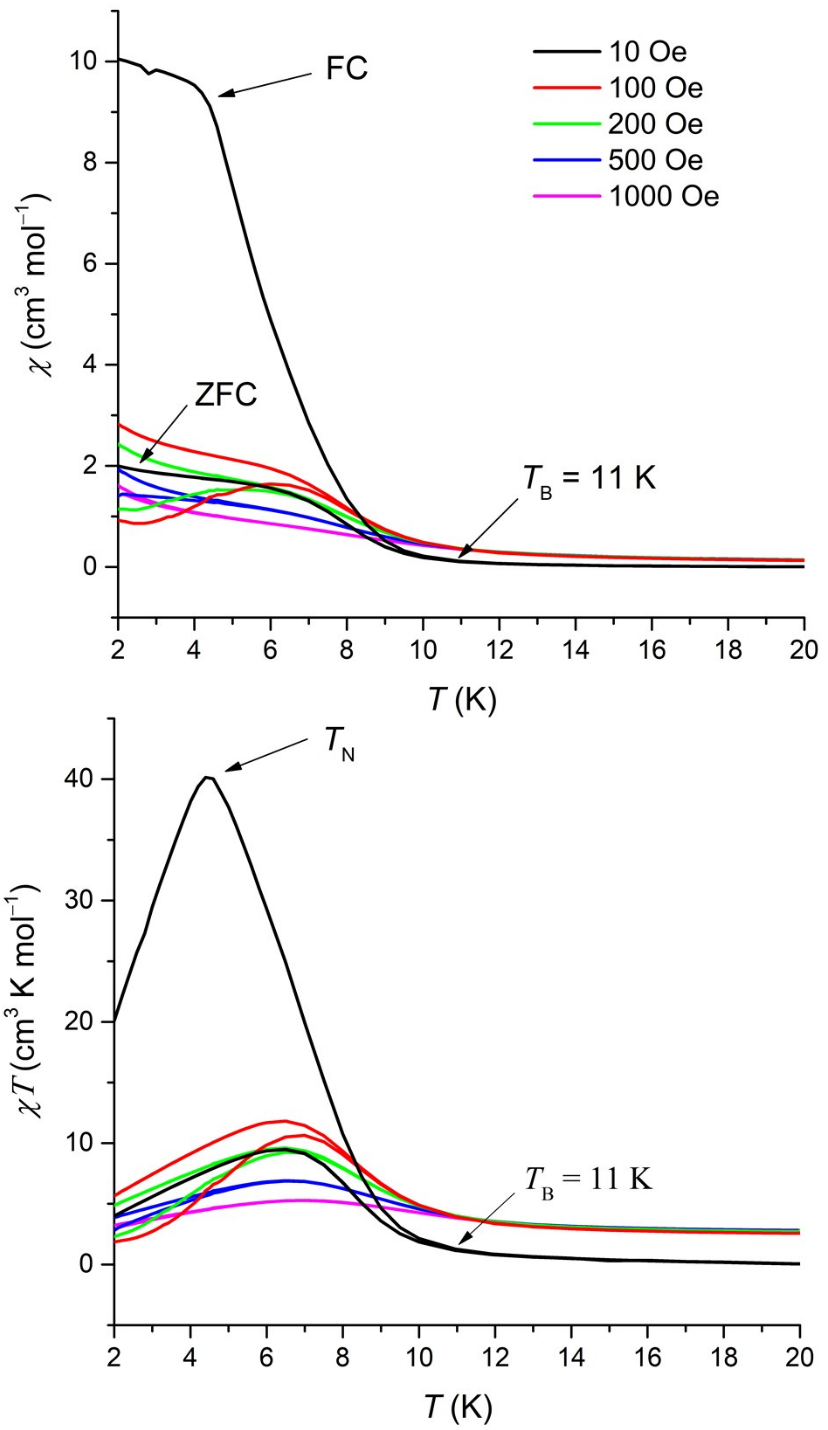

Figure S8. Magnetic susceptibility, χ and magnetic susceptibility temperature product, χT as a function of temperature at 10, 100, 200, 500 and 1000 Oe field in field cooling (FC) and zero field cooling (ZFC) modes.

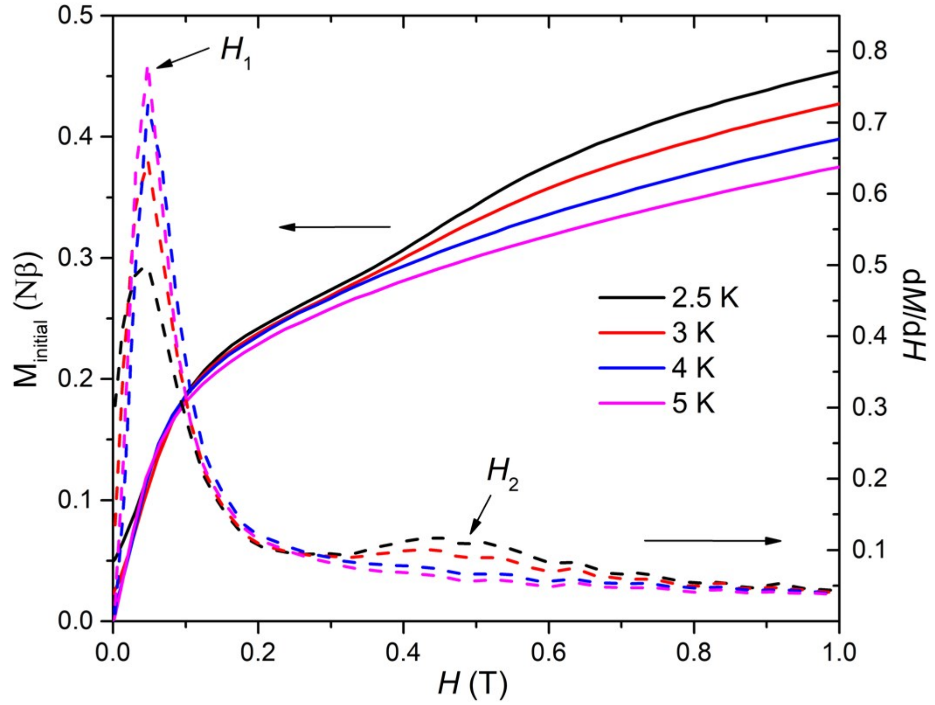

Figure S9. The field dependence of magnetization (M-H curves) in the initial field sweep and its d*M*/d*H* curves measured at 2.5, 3, 4 and 5 K.

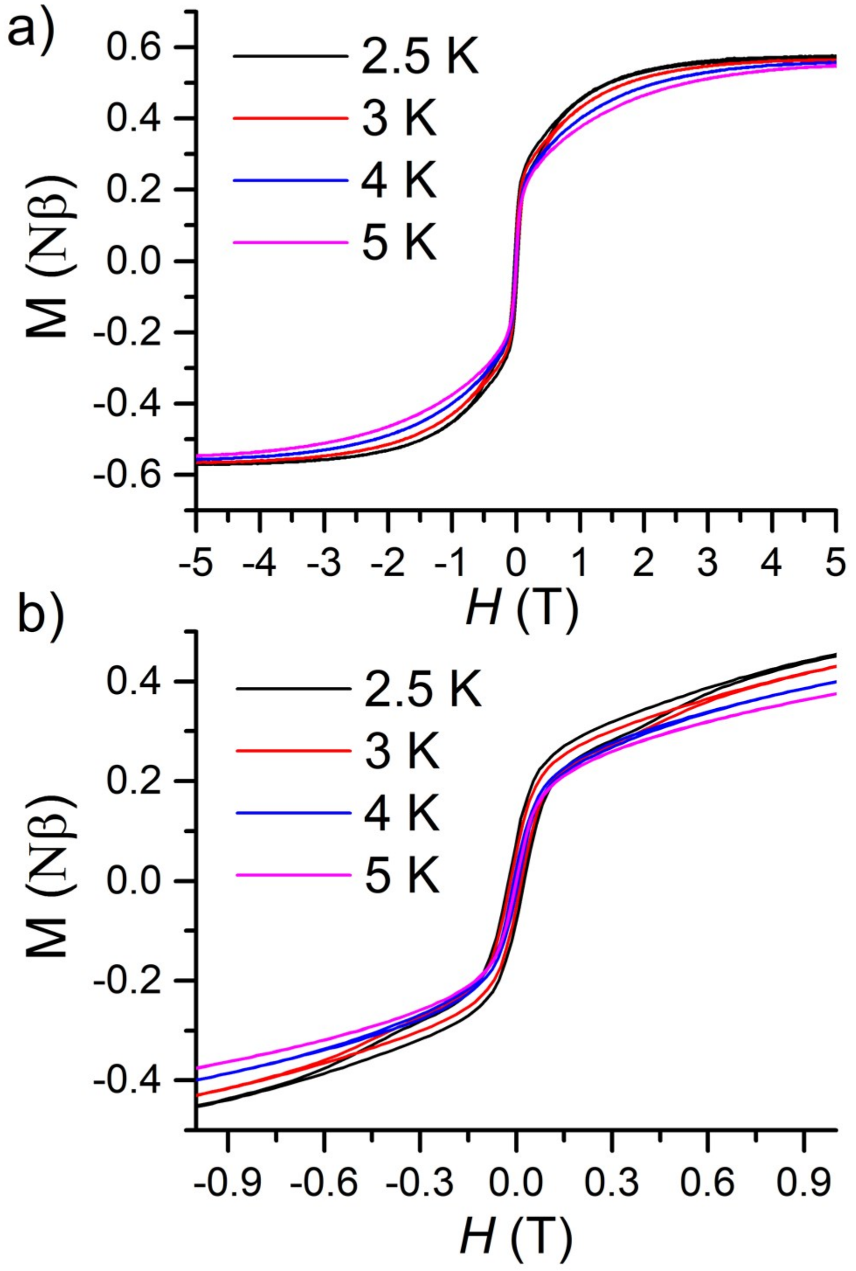

Figure S10. The M-H curves at 2.5, 3, 4 and 5 K, a) M-H in the magnetic field of –5–+5 T, b) Enlarged view of M-H curve in the short magnetic range of –1–+1 T.

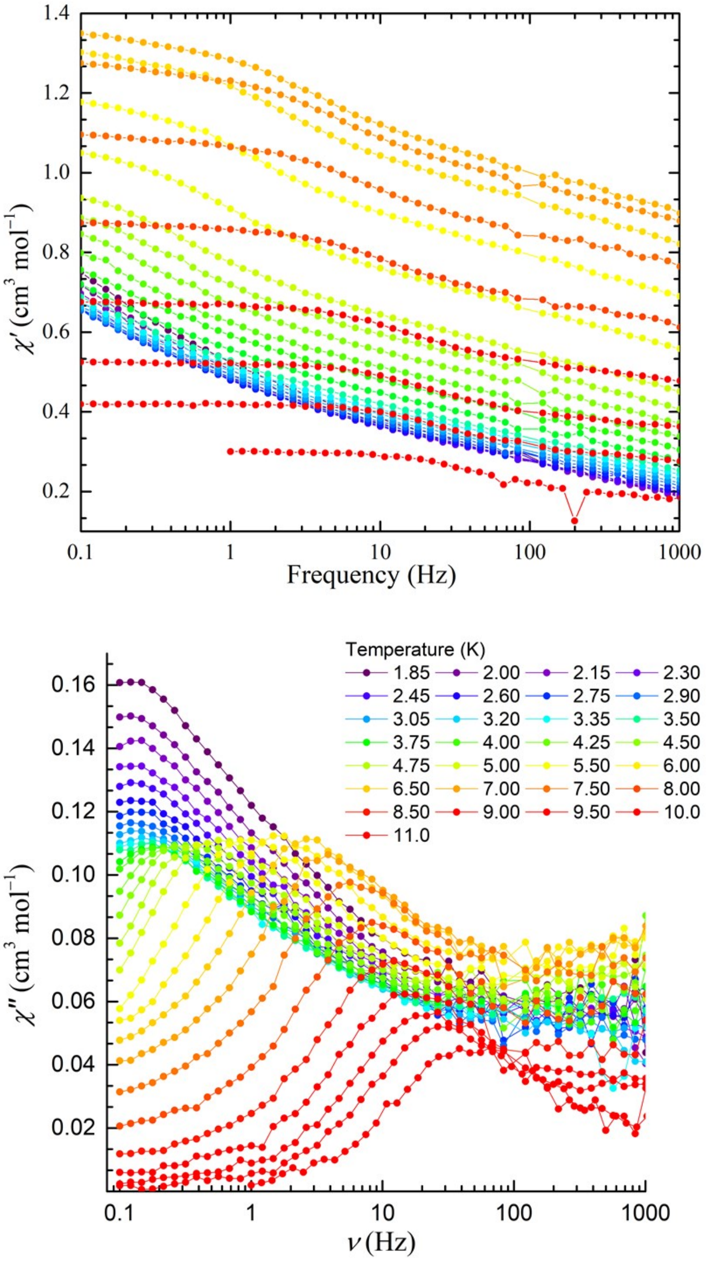

Figure S11. Frequency dependence of in-phase (χ') and out-of-phase magnetic susceptibility (χ'') in the temperature range of 1.85-11.0 K in a 0 Oe field. Below 4 K, the quantum tunnelling of magnetization is dominant, above 4 K, the frequency shifts to a higher region with an increase in the temperature.

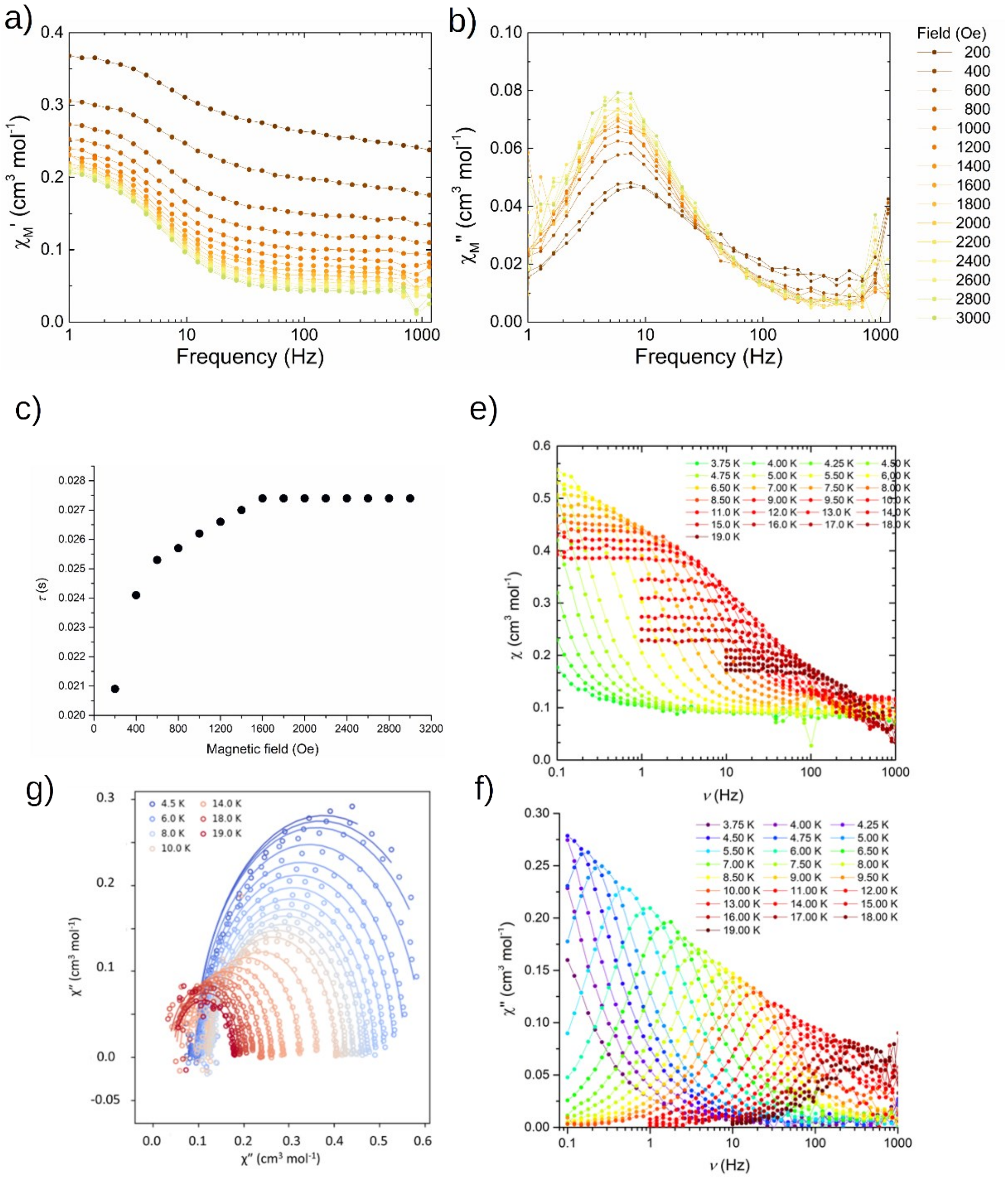

Figure S12. a) Frequency dependence of in-phase magnetic susceptibility (χ') as a function of magnetic field. b) Frequency dependence of out-of--phase magnetic susceptibility (χ'') as a function of magnetic field. c) the plot of relaxation time (τ)-temperature, the τvalues are extracted by using the Havriliak-Negami model, as like:

$$\hat{\chi} = \chi_{adia} + \frac{\chi_{iso1}}{(1 + (i\omega\tau_1)^{1-\alpha_1})^{\beta_1}}$$

e) Frequency dependence of in-phase (χ') in the temperature range of 3.75–19.0 K in a 1500 Oe. f) Frequency dependence of χ'' in the temperature range of 3.75–19.0 K in a 1500 Oe field. g) Cole-Cole plot between χ' and χ'' in a field of 1500 Oe.

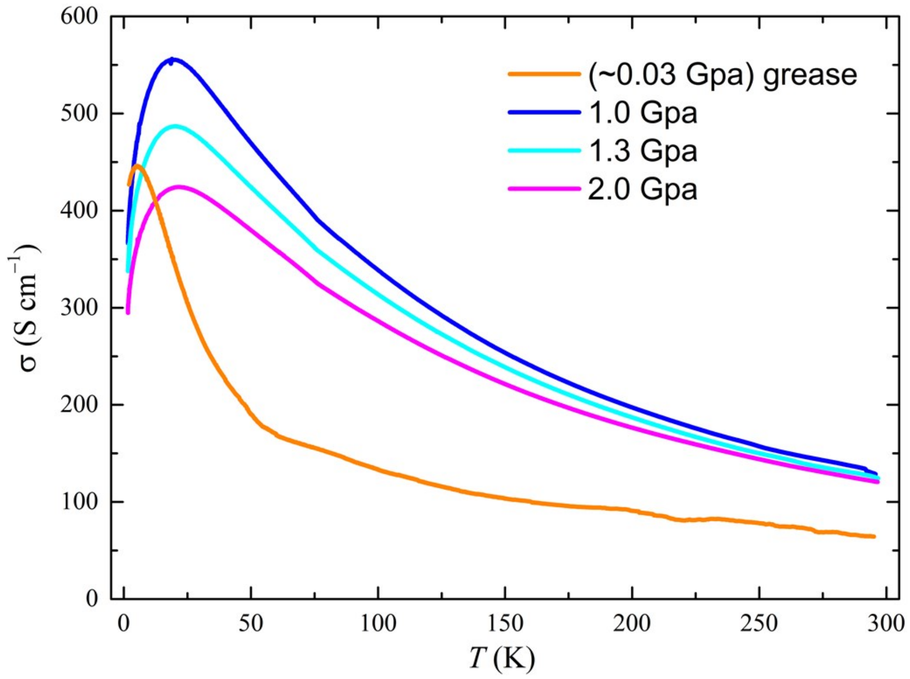

Figure S13. Pressure effects on the electrical conductivity as a function of temperature.

a)

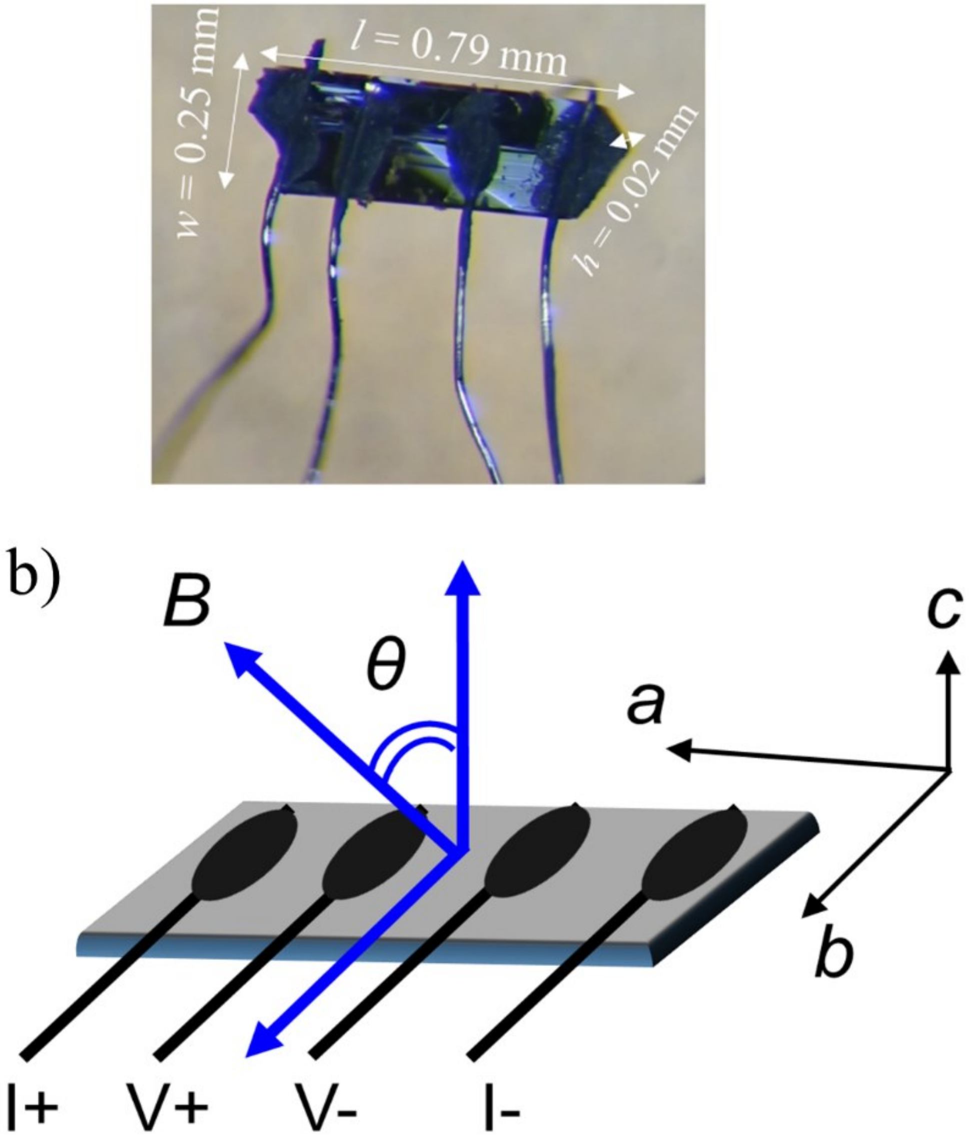

Figure S14. a) Four-probe technique for electrical conductivity measurement with typical size of single crystals. b) The angle determination by adjusting the orientation of the magnetic field and electrical current. The direction of current is parallel to the crystallographic *a*-axis.

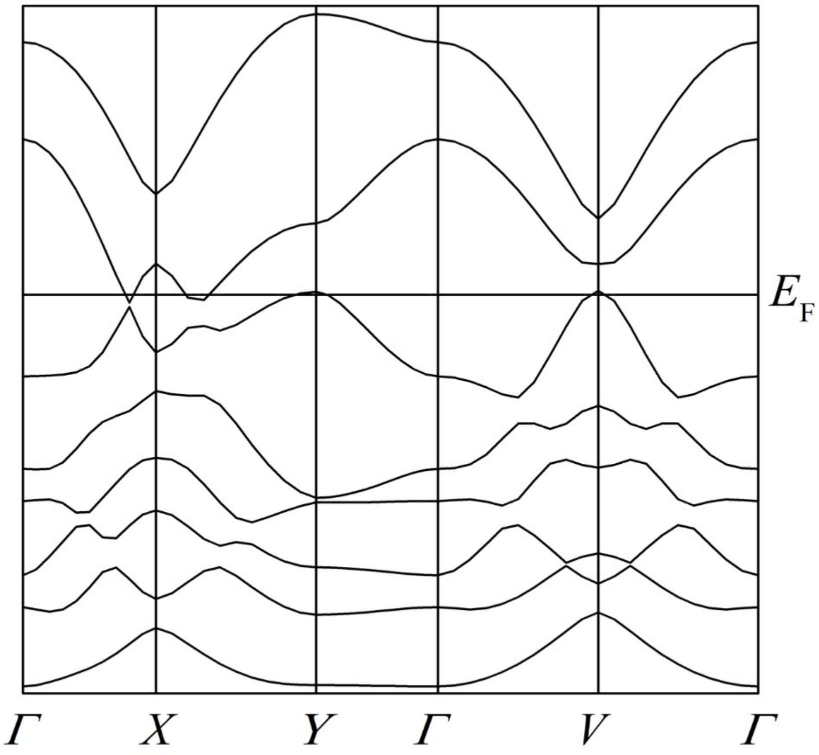

Figure S15. The band structure calculated by extended Hückel method. The high symmetry points are given as *Γ (0, 0, 0) X (0.5, 0 ,0) Y (0, 0.5, 0) V* (0.5, –0.5, 0).

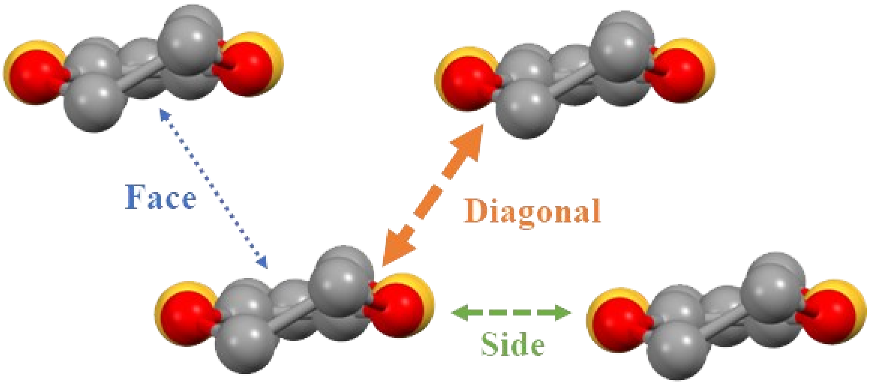

Figure S16. Three different short contacts between adjacent BO units.

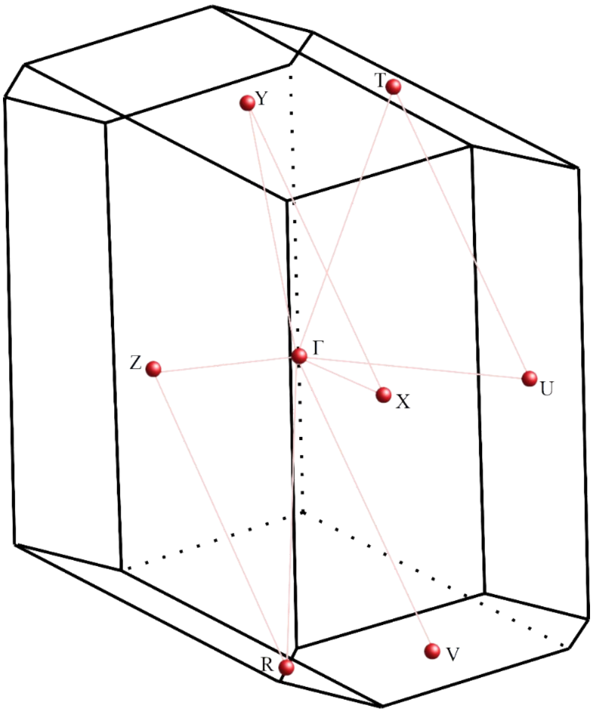

Figure S17. High symmetry points (Γ = (0, 0, 0) X = (0.5, 0, 0) Y = (0, 0.5, 0) Z = (0, 0, 0.5) R = (0.5, –0.5, 0.5) T = (0, 0.5, –0.5) U = (0.5, 0, -0.5) V = (0.5, –0.5, 0) in triclinic $P\bar{}$ space group for the first principle band structure calculation.

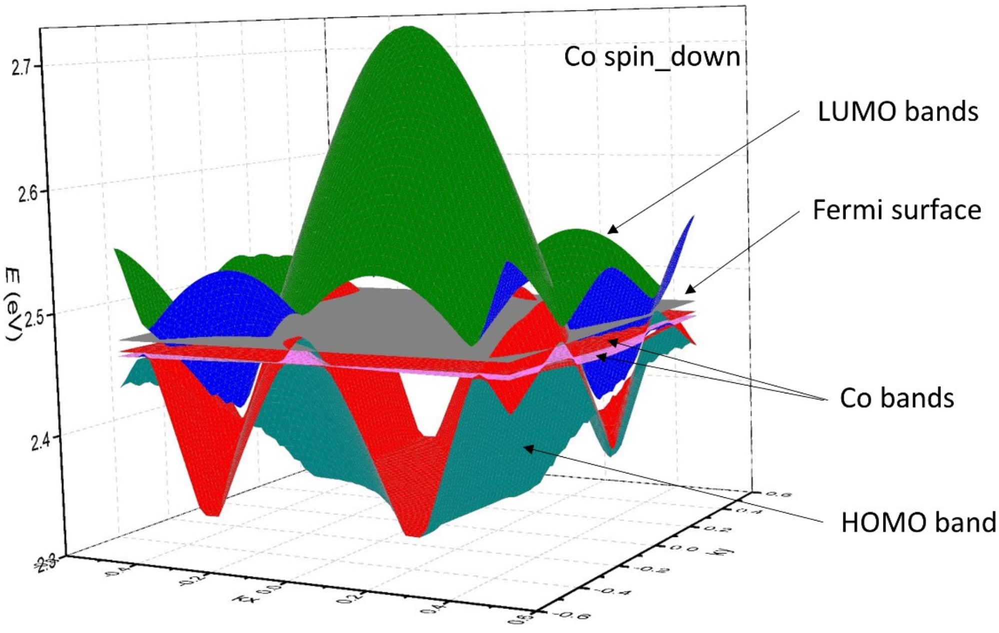

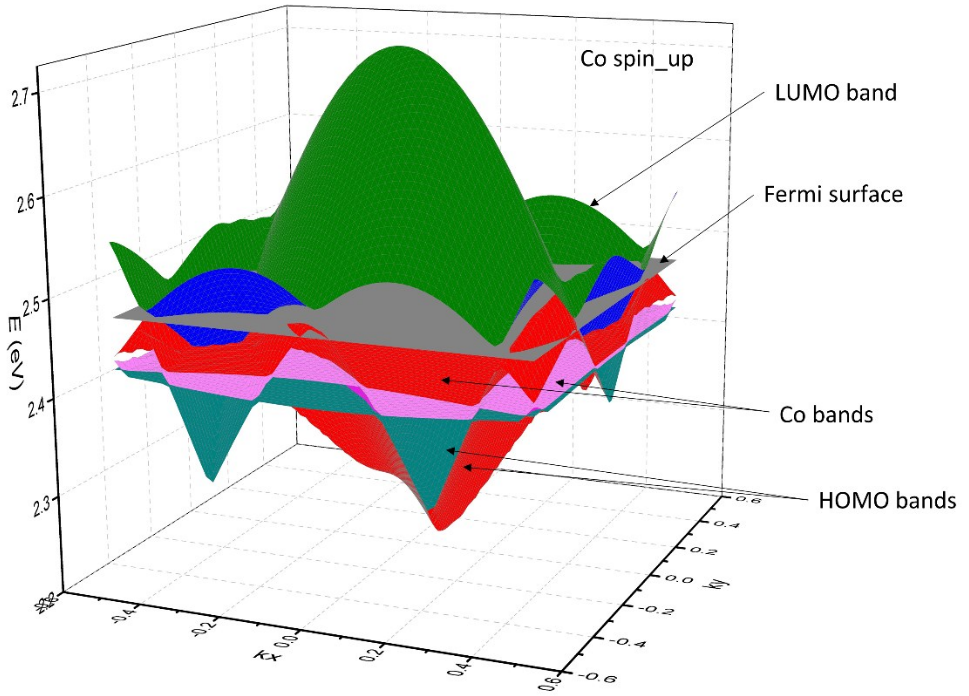

Figure S18. The DFT-based band energy dispersion of **BO4** in Co spin up and down states.

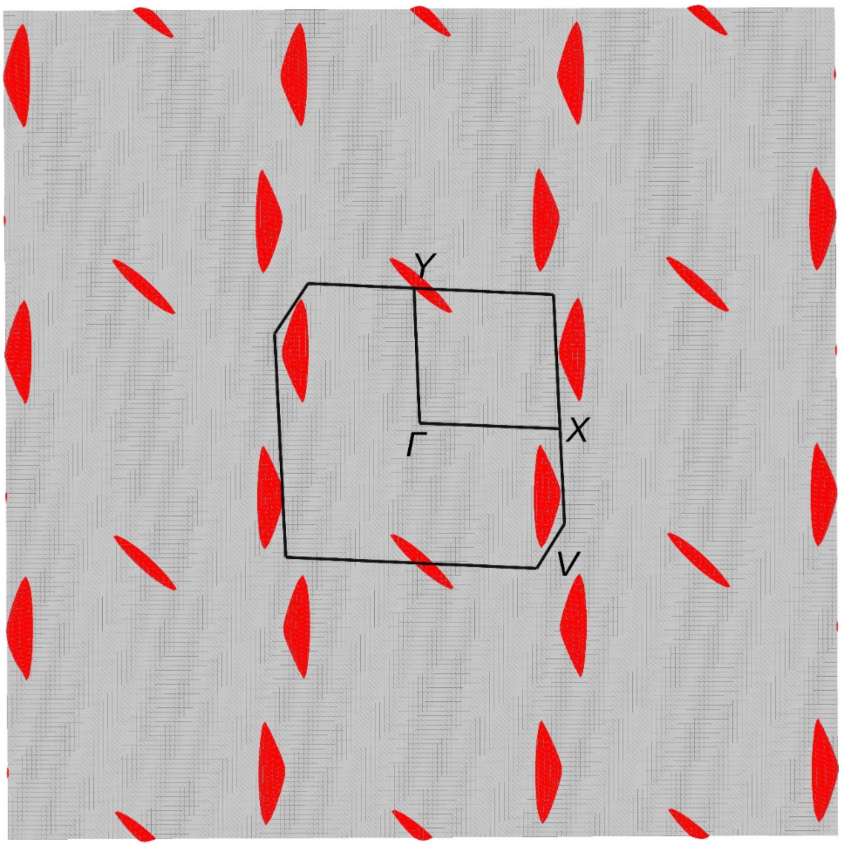

Figure S19. The arrangement of hole tubes in the extended first Brillouin zone by DFT calculation.

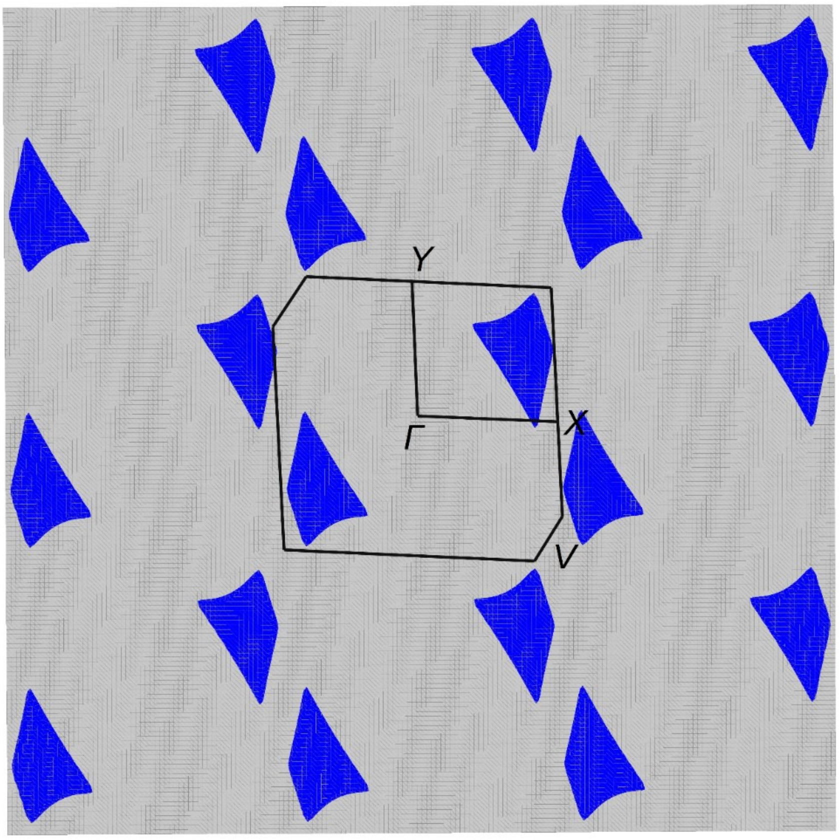

Figure S20. The arrangement of electron tubes in the extended first Brillouin zone by DFT calculation.

Table. S1. Hückel parameters for the band calculation based on the extended Hückel method.

Atom	orbital	H (eV)	[illegible]
S	3s	–20.0	2.12
	3p	–11.0	1.83
	3d	–5.44	1.50
O	2s	–32.3	2.28
	2p	–14.8	2.28
C	2s	–21.4	1.63
	2p	–11.4	1.63
H	1s	–13.6	1.00

Table. S2. Summary of the hydrogen bonds (C-H···O) between layers.

Hydrogen bonds			
Atom1	Atom2	Interatomic distance (Å)	Interatomic distance-vdW (Å)
C11	O25	3.02	–0.20
C29	O27	3.06	–0.16
C40	O26	3.22	–0.01
O19	C31	3.14	–0.08
O22	C12	2.91	–0.32
O24	C9	3.18	–0.04
O24	C10	3.05	–0.17

vdW: the sum of van-der-Waals radii of atom1 and 2

The magnetic susceptibilities for the data between 10–300 K were fitted by using equation (S1):

$$\chi = (1 - \Delta)\frac{\chi_{calc} + \eta}{1 - \left(\frac{zJ}{N_A{\mu_B}^2}\right)(\chi_{calc} + \eta)} + (\Delta)\chi_\Delta \quad \text{(S1)}$$

where Δ is the fraction of magnetic impurity ($\Delta = 0.0026, \chi_\Delta = 1.55 \times 10^{-5}$ emu mol^{-1}), η is the temperature-independent paramagnetism, μ_B is Bohr magneton, N_A is Avogadro's number and zJ is the intermolecular interaction parameter between two Co(pdms)$_2$ units ($z = 2$). $\chi_{(\eta)} = 0.0032$ cm^3 mol^{-1} under ZFC, and $\chi_{(\eta)} = 0.0028$ cm^3 mol^{-1} under FC conditions

References

[1] M. Iyoda, Y. Kuwatani, E. Ogura, K. Hara, H. Suzuki, T. Takano, K. Takeda, J. Takano, K. Ugawa, M. Yoshida, H. Matsuyama, H. Nishikawa, I. Ikemoto, T. Kato, N. Yoneyama, J. Nishijo, A. Miyazaki, T. Enoki, *Heterocycles.* **2001**, *54*, 833-+.

[2] Y. Rechkemmer, F. D. Breitgoff, M. van der Meer, M. Atanasov, M. Hakl, M. Orlita, P. Neugebauer, F. Neese, B. Sarkar, J. van Slageren, *Nat. Commun.* **2016**, *7*.

[3] C. CrystalClear-SM 1.4.0 SP1, Rigaku.

[4] A. Altomare, M. C. Burla, M. Camalli, G. L. Cascarano, C. Giacovazzo, A. Guagliardi, A. G. G. Moliterni, G. Polidori, R. Spagna, *J. Appl. Crystallogr.* **1999**, *32*, 115-119.

[5] G. M. Sheldrick, *Acta Crystallographica a-Foundation and Advances.* **2008**, *64*, 112-122.

[6] G. A. Bain, J. F. Berry, *J. Chem. Educ.* **2008**, *85*, 532-536.

[7] G. Kresse, J. Hafner, *Phys. Rev. B. Condens. Matter.* **1993**, *47*, 558-561.

[8] J. P. Perdew, K. Burke, M. Ernzerhof, *Phys. Rev. Lett.* **1996**, *77*, 3865-3868.

[9] T. Mori, A. Kobayashi, Y. Sasaki, H. Kobayashi, G. Saito, H. Inokuchi, *B. Chem. Soc. Jpn.* **1984**, *57*, 627-633.
